# Prognostic impact of the timing of antihypertensive medication initiation for hypertension detected at health screening on primary prevention of adverse cardiovascular events: Age-stratified real-world data analysis

**DOI:** 10.1038/s41440-025-02249-1

**Published:** 2025-06-19

**Authors:** Hiromasa Ito, Tomohisa Seki, Yoshimasa Kawazoe, Toru Takiguchi, Yu Akagi, Kazumi Kubota, Kana Miyake, Masafumi Okada, Kazuhiko Ohe

**Affiliations:** 1 Department of Healthcare Information Management, The University of Tokyo Hospital, Tokyo, Japan; 2 Department of Cardiology and Nephrology, Mie University Graduate School of Medicine, Mie, Japan; 3 Artificial Intelligence and Digital Twin in Healthcare, Graduate School of Medicine, The University of Tokyo, Tokyo, Japan; 4 Department of Emergency and Critical Care Medicine, Nippon Medical School, Tokyo, Japan; 5 Department of Biomedical Informatics, Graduate School of Medicine, The University of Tokyo, Tokyo, Japan

**Keywords:** Antihypertensive agents, Implemental hypertension, Primary prevention, Proportional hazards models, Real-world data

## Abstract

The association between age and timing of antihypertensive treatment initiation and its effect on outcomes of patients with hypertension remain unclear. We investigated the impact of the time to antihypertensive therapy initiation for cardiovascular event primary prevention in an age-stratified analysis using data from a nationwide health claims database. This observational cohort study analyzed claim and health examination data recorded between January 1, 2005, and April 30, 2021, in the Japan Medical Data Center database. Patients with hypertension treated with antihypertensive agents were grouped by time (years) to therapy initiation: <1 (reference group), 1–2, and ≥2. The primary outcome was a composite outcome encompassing cardiovascular death, acute coronary syndrome, heart failure, and cerebrovascular disease. The secondary outcome was all-cause mortality. Cox proportional hazard models were used to calculate hazard ratios and 95% confidence intervals adjusted for the time to treatment (TTI) group, age, male sex, systolic blood pressure, smoking status, dyslipidemia, diabetes, and visceral obesity. Among 520,669 participants, TTI ≥ 1 year conferred significantly higher hazard ratios for primary outcomes than TTI < 1 year in individuals aged ≥40 years. Hazard ratios (95% confidence intervals) for the primary outcome with TTI of 1–2 and >2 years were 1.215 (1.073–1.375) and 1.296 (1.163–1.444) in those aged 40–49 years and 1.268 (1.144–1.406) and 1.341 (1.224–1.468) in those aged 50–59 years, respectively. TTI ≥ 2 years was an independent prognostic factor for the secondary outcome of all-cause mortality in those aged ≥40 years.

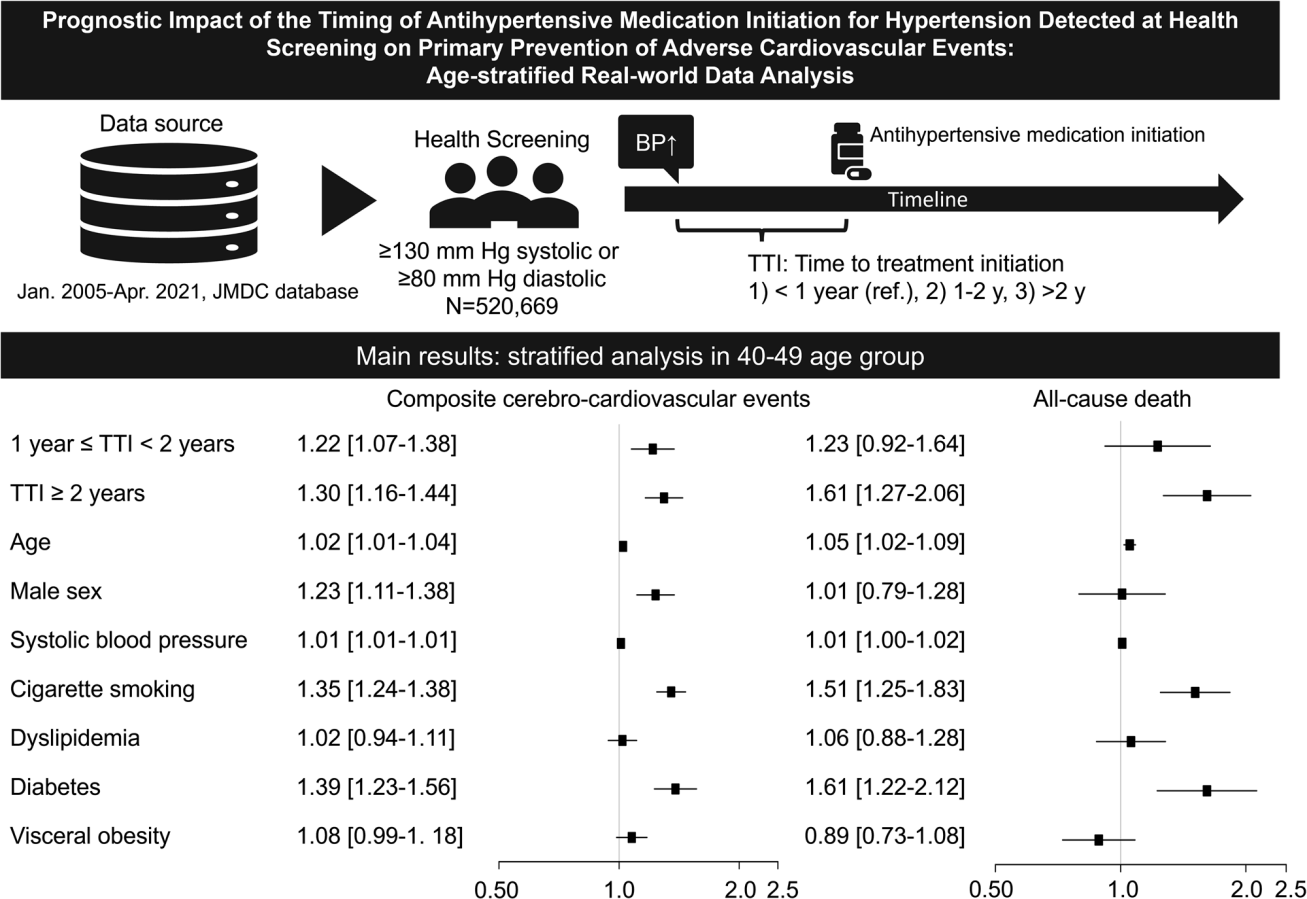

## Introduction

Hypertension represents a significant global public health challenge and is widely recognized as a condition that markedly elevates the risk of cerebrovascular and cardiovascular diseases, including stroke, myocardial infarction, heart failure, and chronic kidney disease [1–6]. In Asia, including Japan, the increasing prevalence of hypertension, attributable to lifestyle changes and population aging, underscores the urgency of early diagnosis and timely therapeutic intervention [7–9]. Public and occupational health examinations are conducted to facilitate the early identification of individuals at risk of hypertension. For patients with hypertension, the next step involves individual risk profile-based customization of treatment strategies.

Major international guidelines on hypertension, such as the 2017 American College of Cardiology (ACC)/ American Heart Association (AHA) guideline and 2024 European Society of Cardiology (ESC) guidelines, recommend immediate pharmacotherapy initiation in older adults and patients with a high risk of atherosclerotic cardiovascular disease, identified through risk assessment tools [10–13]. However, the optimal age for initiating pharmacotherapy in younger patients or those with low-to-moderate risk of cardiovascular events remains unknown. This constitutes a knowledge gap regarding the age-appropriate timing of pharmacotherapy initiation in non-elderly and relatively low-risk patients with hypertension. However, the duration of untreated hypertension is associated with the occurrence of cardiovascular events [14], and to optimize treatment strategies, it is important to understand how age and time to treatment initiation affect cardiovascular outcomes.

This study aimed to assess the impact of the time to initiation of antihypertensive therapy on the occurrence of cardiovascular events by age-stratified analysis using data from a nationwide health claims database.

## Methods

### Study design

This retrospective, observational cohort study utilized data from the Japan Medical Data Center (JMDC) Claims Database, which contains medical claims and health examination data in Japan [15–20]. The JMDC database is a commercially available, anonymized health insurance claims database in Japan. It primarily includes data from employees and their dependents covered under employment-based health insurance schemes. The database captures medical diagnoses, procedures, and prescription records using standardized formats [21]. This database, available for purchase from JMDC, contains data of approximately 11.6 million individuals recorded from January 1, 2005, through April 30, 2021, that includes diagnostic information based on the International Classification of Diseases, Tenth Revision (ICD-10), prescription data based on the Anatomical Therapeutic Chemical (ATC) classification system, medical practice details based on electronic claims processing codes, and results of certain medical examinations.

This study was conducted in accordance with the STROBE guidelines for reporting observational studies. This study conformed to the principles of the Declaration of Helsinki and was approved by the Ethics Committee of the University of Tokyo Hospital (Approval No. 2024278NIe). This study used anonymized data, and therefore, the requirement for obtaining informed consent from participants was waived.

### Participants

Of the 6 196 059 unique records with measurements of blood pressure (BP), 2,517,700 records with normal BP ( < 120 mm Hg systolic and <80 mm Hg diastolic), and 543,820 records with high normal BP (120–129 mm Hg systolic and <80 mm Hg diastolic) were excluded. Records of those aged <20 years at the time of health screening (*n* = 149,502), those not using antihypertensive agents (*n* = 2 323 205), those with cardiovascular or renal events (*n* = 227,339), and those with events occurring before treatment with antihypertensive medications (*n* = 43,466) were further excluded. The final study cohort comprised 520,669 participants. The inclusion criteria were age ≥20 years at the time of health screening with the classification of elevated BP and grade I hypertension ( ≥ 130 mm Hg systolic or ≥80 mm Hg diastolic) identified in health screening data and no history of cerebro-cardiovascular events before using antihypertensive agents. The date of the first hypertension diagnosis was when the BP level exceeded the criterion value for the first time at a medical examination recorded in the database.

### Procedures

In the Japanese health examination system, BP was measured by healthcare professionals in accordance with the protocol recommended by the Japanese Ministry of Health, Labor and Welfare. Measurements were taken on the right arm using a standard sphygmomanometer or an automated device after participants had rested for 5 min in a seated position (see Appendix p 2) [20].

There are differences in the BP thresholds for diagnosis and treatment initiation among major hypertension guidelines, including those in Japan [22]. This study primarily followed the guidelines of the Japanese Society of Hypertension when hypertension and the criteria for treatment initiation were defined and determined, respectively. We defined (1) the elevated BP group as those with SBP 130–139 mm Hg or DBP 80–89 mm Hg and (2) the grade I hypertension group as those with SBP ≥ 140 mm Hg or DBP ≥ 90 mm Hg [8]. Similarly, body mass index, waist circumference, and fasting laboratory data were obtained from the JMDC database. Information on cigarette smoking and history of diseases were self-reported [23]. Visceral obesity was defined as waist circumference ≥85 cm for men and ≥90 cm for women [24]. Overweight was defined as body mass index ≥25 kg/m^2^ [25]. Diabetes was defined as a fasting glucose level of ≥126 mg/dL or the use of glucose-lowering medications. Dyslipidemia was defined as low-density lipoprotein cholesterol levels ≥140 mg/dL, high-density lipoprotein cholesterol levels <40 mg/dL, triglyceride levels ≥150 mg/dL, or the use of lipid-lowering medications [20].

Time to treatment initiation (TTI) was defined as the period between the time point when hypertension was indicated in the health check-up and the time point when antihypertensive medication was used. Based on the TTI, patients were divided into three groups: <1 (reference)1–2, and ≥2, years.

The prescription of antihypertensive agents was identified using the ATC codes (see Appendix p 3). The prescription of antihypertensive medication was determined by the presence of prescription records in the database within a specified period after the health examination. Antihypertensive medications and agent combinations were summarized descriptively for up to three lines of treatment per patient in the sunburst plot. Sankey diagrams were used to depict hypertensive pharmacotherapy sequences to illustrate a flow from one set of values to another. These diagrams do not reflect the duration of treatment or the timing of switching treatments.

### Outcomes

The primary endpoint comprised the following composite outcomes requiring hospitalization: cardiovascular death, acute coronary syndrome (ICD-10: I200, I21, I22), heart failure (ICD-10: I110, I130, I132, I50), and cerebrovascular disease (ICD-10: I60, I61, I62, I63, I64). Cerebrovascular disease was defined as including cerebral infarction (ICD-10: I63, I64), cerebral hemorrhage (ICD-10: I61, I62), and subarachnoid hemorrhage (ICD-10: I60). Events were defined as including cases where an ICD-10 diagnosis code was recorded with diagnosis procedure combination (DPC) claim within the same month. Cardiovascular death was defined as an inpatient case with a primary diagnosis of cardiovascular disease (ICD-10: I00–I99) and a recorded discharge status of death in hospitalization receipt or DPC data. The validity of using ICD-10 codes from DPC claims as outcome definitions has been confirmed in validation studies using JMDC data, demonstrating high positive predictive values for myocardial infarction, stroke, and heart failure [26, 27]. In addition, a previous study using the JMDC database examined cardiovascular events requiring hospitalization as primary outcome [28].

The secondary outcome was all-cause death. Deaths were based on hospitalization or DPC records. Outcome occurrence was defined as the time the event was first recorded, and for participants with multiple events, only the first event was considered as an outcome. The frequency of follow-up was based on each month, which is the unit of the receipt data.

### Statistical analysis

Continuous variables with normal distributions, confirmed by the histogram and Q-Q plot, are expressed as mean (standard deviation) values. Medians (interquartile ranges) were given for the data with skewed distribution. Categorical variables are expressed as frequency (proportion) unless otherwise indicated. Baseline characteristics were compared with the chi-square or Fisher’s exact test for categorical variables; parametric data were analyzed using unpaired Student’s t-test with Welch’s correction when appropriate, while non-parametric data were analyzed using the Mann-Whitney U test. Kaplan-Meier curves were used to assess the cumulative incidence of primary outcome within each TTI group. The cumulative incidence of each event was calculated using the model for the subdistribution of a competing risk [29].

The time variable in the Cox proportional hazards model was adjusted to isolate the effects of early initiation of medication and duration of exposure to hypertension on the effect. Time zero was set at the first detection of high BP, and left truncation was introduced, where entry time was at treatment initiation and exit time was at the event or censoring. Previous studies have shown that ignoring left truncation can lead to biased survival estimates and distorted hazard ratios. Adjusting for left truncation has been recommended as an effective way to correct for delayed entry and avoid immortal time bias in real-world data [30, 31]. Using this approach, Cox regression analyses were performed to assess the association of TTI groups with subsequent risk for outcomes. Hazard ratios (HRs) of outcomes were calculated in an unadjusted model and after adjusted by TTI group, age, male sex, systolic BP, habitual cigarette smoking, history of diabetes, history of dyslipidemia, and visceral obesity after confirming the assumptions of the proportionality of hazards by examinations of Schoenfeld residuals. Covariates were selected based on the components of metabolic syndrome and factors contributing to the occurrence of cardiovascular events [24, 32]. The correlation coefficients for each covariate are presented in the appendix (p 4). As TTI is highly dependent on the participants’ age, a stratified analysis was performed for 10-year intervals, starting from the age at the time of health screening. In the main analyses, a complete case analysis was performed, excluding individuals with missing values in any of the covariates included in the respective models.

To confirm the robustness of the results, seven sensitivity analyses were performed. First, the analysis was performed after restricting the study population to patients with elevated BP. Second, the analysis was performed after restricting the study population to patients with grade I or higher hypertension. Third, the cohort was limited to participants who had undergone at least two health check-ups, with a focus on those who were not identified as hypertension at the first check-up but were newly diagnosed with hypertension at or after their second check-up. This approach excluded cases of pre-existing hypertension before enrollment in the database, which facilitated a more precise definition of the duration of hypertension. Fourth, in the Cox proportional hazards model, instead of visceral obesity, overweight, defined by BMI, was used as a covariate. Fifth, we used multiple imputation for missing data by predictive mean matching using the R package mice [33]. Twenty imputations were performed, with each imputed dataset generated over 50 iterations. Predictive mean matching was chosen because it preserves the original data distribution and is robust to the imputation of continuous variables. Sixth, to account for the potential influence of early events, a Cox proportional hazards analysis was conducted excluding events that occurred within the first year after treatment initiation. Seventh, Fine-Gray modeling was used as a competing risk analysis in this study, as deaths other than cardiovascular deaths can be considered as competing risks with primary events. Significance was defined at *p* < 0.05, and statistical analyses were performed using R version 4.3.3 (R Core Team, Vienna, Australia).

### Data sharing

The relevant analytical protocol and code underlying this analysis are available upon request to the corresponding author (HI). The JMDC database is a commercial database and cannot be accessed through this request. This database is available for purchase from JMDC Inc (Tokyo, Japan).

## Results

### Baseline characteristics

**Fig. 1:**
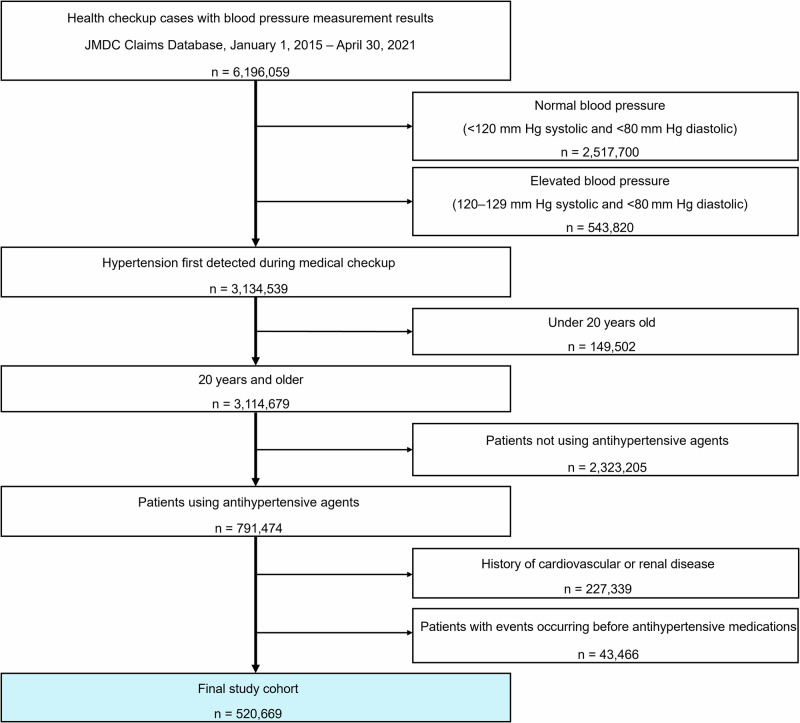
Flowchart depicting the cohort study design and patient screening and enrollment. JMDC Japan Medical Data Center

Data from a total of 520,669 participants were included in the analysis (Fig. 1). Table 1 shows details of participant characteristics, stratified by TTI into three groups. Details of the characteristics by age group are shown in the appendix (p 5–11). The patients’ average age was 53.1 years, and 70.9% were male. In the group with TTI less than 1 year, the stratified analysis revealed that the mean age was statistically higher, with a lower proportion of participants aged ≤40 years and a higher proportion aged ≥60 years. BP tended to be higher in the group with TTI between 1 and 2 years. Calcium channel blockers and angiotensin II receptor blockers were the most commonly prescribed. The detailed utilization patterns are visually represented using a sunburst plot and Sanky plot. The sunburst plot is available in the public repository (https://hiromaito.github.io/sunburstplot-repository/sunburstplot.html), and the Sankey diagram is shown in the appendix (p 12).

**Table 1:** Participants’ characteristics stratified by time to treatment initiation

		Time to treatment initiation (TTI), years			*P*-value	Std diff
Variable	Missing	<1	1–2	≥2		
*n*		357,827	49,510	118,672		
Age, years, *n* (%)	0	54.87 (8.50)	50.82 (8.71)	48.59 (8.13)	<0.001	0.497
20–29	0	1402 (0.39)	559 (1.13)	1452 (1.22)		
30–39	0	11,847 (3.31)	3776 (7.63)	11,384 (9.59)		
40–49	0	80,490 (22.49)	17,518 (35.38)	53,184 (44.82)		
50–59	0	150,353 (42.02)	19,110 (38.60)	40,765 (34.35)		
≥60	0	113,735 (31.78)	8547 (17.26)	11,887 (10.02)		
Male sex, *n* (%)	0	246,779 (68.97)	34,342 (69.36)	88,230 (74.35)	<0.001	0.080
Body mass index, kg/m^2^	228	25.53 (4.36)	24.97 (4.20)	24.70 (3.95)	<0.001	0.132
Overweight, *n* (%)	228	178,207 (49.80)	21853 (44.14)	49167 (41.43)	<0.001	0.113
Waist circumference, cm	9231	88.56 (10.78)	86.79 (10.48)	86.03 (9.92)	<0.001	0.161
Visceral obesity, *n* (%)	9231	198,400 (55.45)	24,057 (48.59)	54,158 (45.64)	<0.001	0.163
Systolic blood pressure, mmHg	0	138.96 (14.98)	141.50 (15.38)	136.75 (13.21)	<0.001	0.218
Diastolic blood pressure, mmHg	0	87.05 (9.89)	89.89 (10.21)	87.43 (8.70)	<0.001	0.194
Stage 1 hypertension, *n* (%)	0	181,995 (50.86)	19,775 (39.94)	63,886 (53.83)	<0.001	0.187
Stage 2 hypertension, *n* (%)	0	175,832 (49.14)	29,735 (60.06)	54,786 (46.17)	<0.001	0.187
Triglycerides, mg/dL	2504	113 (79–165)	109 (75–162)	108 (75–163)	<0.001	0.015
HDL cholesterol, mg/dL	2306	60.54 (16.75)	60.84 (16.95)	59.94 (16.35)	<0.001	0.036
LDL cholesterol, mg/dL	2672	123.39 (30.50)	129.28 (32.74)	128.27 (31.97)	<0.001	0.125
AST, U/L	2174	26.54 (15.13)	25.39 (14.36)	24.70 (13.00)	<0.001	0.086
ALT, U/L	2157	29.97 (22.76)	28.87 (23.02)	28.77 (22.52)	<0.001	0.035
γ-glutamyl transpeptidase, IU/L	2457	36 (23–63)	34 (22–60)	34 (22–59)	<0.001	0.045
Fasting blood glucose, mg/dL	105,642	100 (92–112)	96 (90–105)	95 (89–103)	<0.001	0.206
HbA_1c_, %	60,417	5.86 (0.87)	5.71 (0.87)	5.62 (0.74)	<0.001	0.192
Hemoglobin, g/dL	77,676	14.70 (1.42)	14.79 (1.53)	14.81 (1.53)	<0.001	0.052
Cigarette smoking, *n* (%)	427	88,876 (24.84)	15,190 (30.68)	39714 (33.47)	<0.001	0.127
Diabetes, *n* (%)	77,896	40,389 (11.29)	3237 (6.54)	5448 (4.59)	<0.001	0.169
Dyslipidemia, *n* (%)	3198	178,440 (49.87)	26,225 (52.97)	61,621 (51.93)	<0.001	0.055
Calcium channel blockers	0	281,648 (78.71)	37,620 (75.98)	89,583 (75.49)	<0.001	0.051
Beta blockers	0	54,942 (15.35)	7617 (15.38)	15,600 (13.15)	<0.001	0.043
Angiotensin-converting enzyme inhibitors	0	18,720 (5.23)	2510 (5.07)	5238 (4.41)	<0.001	0.025
Angiotensin II receptor blockers	0	251,261 (70.22)	29,829 (60.25)	69,721 (58.75)	<0.001	0.161
Direct renin inhibitors	0	665 (0.19)	52 (0.11)	61 (0.05)	<0.001	0.026
Angiotensin-receptor neprilysin inhibitor	0	43 (0.01)	12 (0.02)	23 (0.02)	0.038	0.006
Antihypertensive diuretics	0	41,427 (11.58)	4180 (8.44)	7893 (6.65)	<0.001	0.115
Alpha-adrenergic agents	0	136 (0.04)	23 (0.05)	40 (0.03)	0.470	0.004
Alpha blockers	0	14,456 (4.04)	1665 (3.36)	3103 (2.61)	<0.001	0.053

Categorical variables are expressed as frequency (proportion) unless otherwise indicated. Continuous variables with normal distributions are shown as mean (standard deviation), and medians (interquartile ranges) were shown for the data with skewed distribution

*HDL* high-density lipoprotein, *LDL* low-density lipoprotein, *AST* aspartate aminotransferase, *ALT* alanine aminotransferase. Baseline characteristics were compared with the chi-square or Fisher’s exact test for categorical variables, parametric data were analyzed using unpaired Student’s t-test with Welch’s correction when appropriate, while non-parametric data were analyzed using the Mann-Whitney U test

### Association between TTI and cardiovascular outcomes

During a mean follow-up period of 1 061 ± 858 days, 48,440 primary endpoint events and 3050 deaths occurred. Detailed cumulative incidence rates for the events included in the primary endpoint can be found in the appendix (p 13). Supplementary Fig. 3 shows the Kaplan-Meier curves for the primary outcome for each TTI group. The cumulative incidence was significantly higher in those aged ≥40 years with a TTI of ≥1 year compared to those with a TTI of <1 year.

**Fig. 2:**
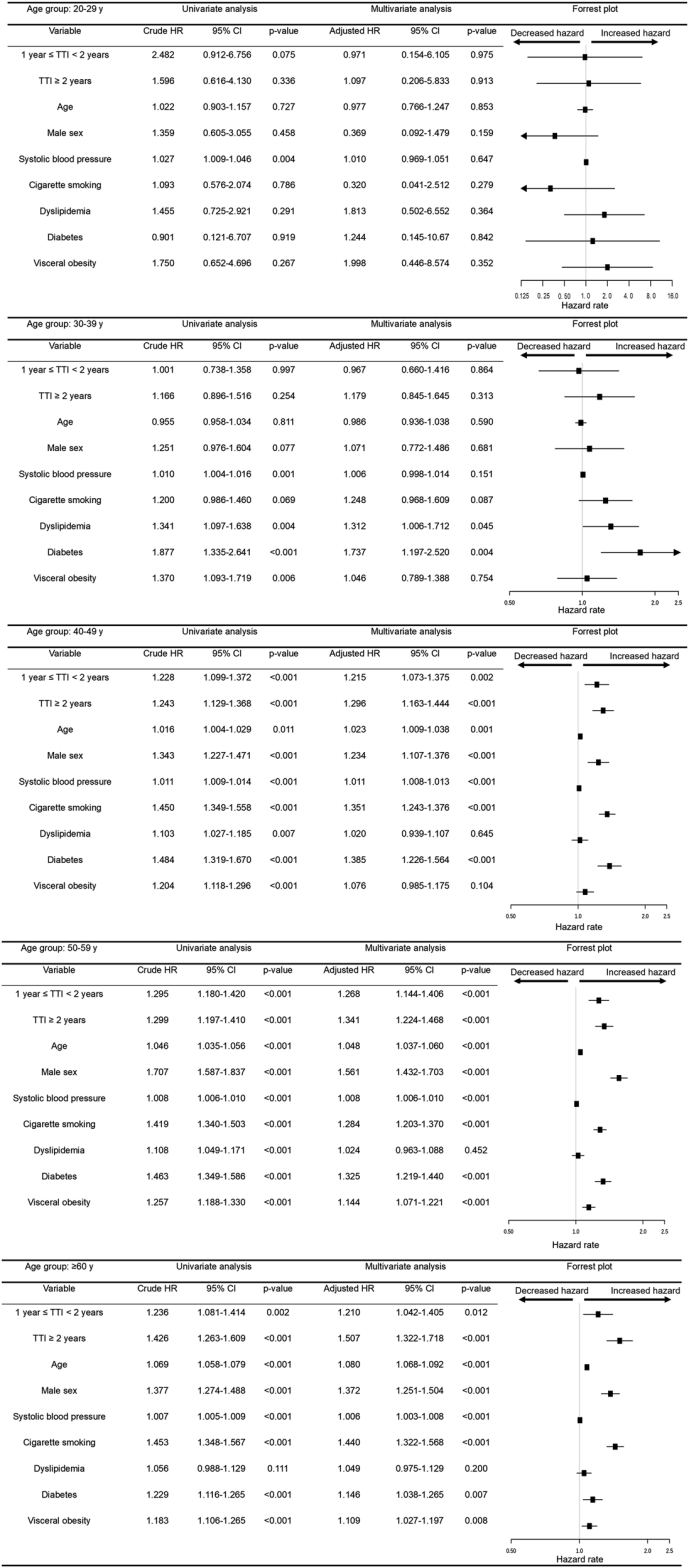
Univariate and multivariate Cox proportional hazard model for primary outcomes. The hazard ratios for each group were calculated using a TTI < 1 year as the reference. The analysis was stratified by age in 10-year increments starting at 20 years, with individuals aged 60–75 years grouped into a single category. Multivariable analysis was adjusted for TTI groups, age, male sex, systolic blood pressure, smoking status, dyslipidemia, diabetes, and visceral obesity. HR hazard ratio, CI confidence index, TTI time to treatment initiation

**Fig. 3:**
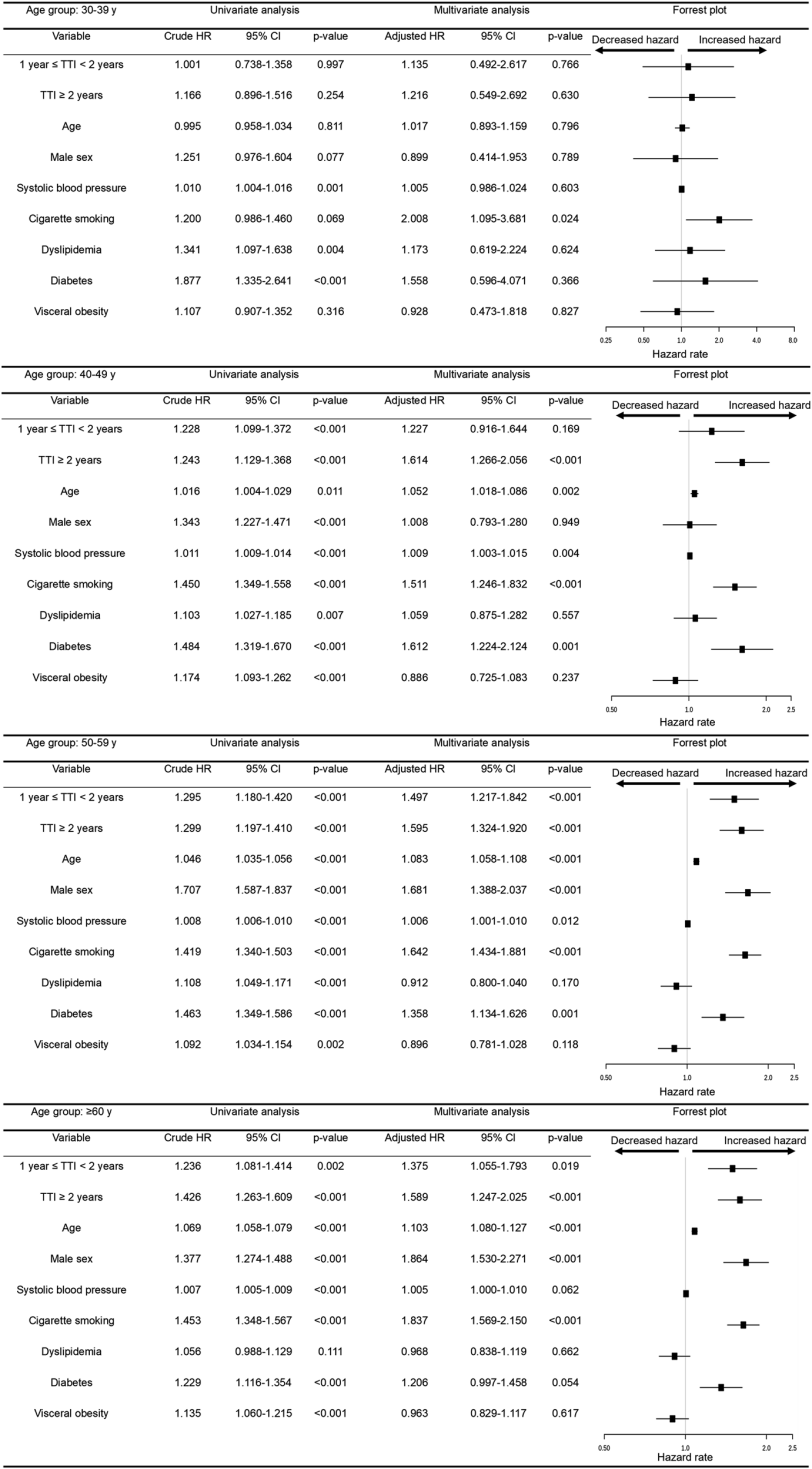
Univariate and Multivariate Cox proportional hazard model for all-cause death. Using the same method of analysis as described in Fig. 2, we found that in participants in the 20–29-year age group, the model could not estimate hazard ratios because of extremely low event counts. HR hazard ratio, CI confidence index, TTI time to treatment initiation, NA not available

The Cox regression analyses for predictors of primary outcomes are summarized in Fig. 2. In the stratified age groups of ≥40 years, as demonstrated by the multivariable analysis and the forest plot of hazard ratios, the group with a TTI of ≥1 year had a statistically significantly higher HR compared to the group with a TTI of <1 year (*p* = *0.002*). In contrast, this trend was not observed in the group with individuals in their 20 s and 30 s (Fig. 2). In the 40–49-year age group, the HRs for the primary outcome were 1.215 (95% CI, 1.073–1.375) for TTI of 1–2 years and 1.296 (95% CI, 1.163–1.444) for TTI ≥ 2 years. In the 50–59-year age group, the HRs were 1.268 (95% CI, 1.144–1.406) and 1.341 (95% CI, 1.224–1.468); in the >60-year age group, the HRs were 1.210 (95% CI, 1.042–1.405) and 1.507 (95% CI, 1.322–1.718), respectively. Additionally, in the ≥40-year age group, age, male sex, SBP, cigarette smoking, and diabetes were identified as common factors associated with an increased risk of composite cerebro-cardiovascular events. Figure 3 shows the results of the Cox proportional hazards analysis evaluating the impact on all-cause mortality. In the 40–49-year age group, TTI of ≥2 years was associated with an adverse prognosis. Furthermore, in those aged ≥50 years, TTI of ≥1 year was identified as an independent prognostic factor for all-cause mortality. Due to the extremely small number of deaths (less than 5) in the 20–29-year age group, the results of the Cox proportional hazards analysis were not shown.

### Sensitivity analyses

The results of six sensitivity analyses are shown in the appendix. The results of the Cox proportional hazards model for the cohort of patients with elevated BP (*N* = 256,906), grade I hypertension (*N* = 251,603), and the cohort of individuals diagnosed with hypertension for the first time at their second or subsequent health examination (*N* = 93,724) are shown in the appendix (p 15–23). In these cohorts, the results were consistent with those of our main analysis. Similar results were observed when the covariate for visceral obesity, defined by waist circumference, was replaced by overweight, defined by BMI (the appendix p 24–26). In the Cox proportional hazards model using multiple imputation, TTI of ≥1 year significantly influenced the increased hazard in all age groups (p 27–29). In the sensitivity analysis excluding events within the first year after treatment initiation, the trend of increasing HRs with age remained evident in the group with TTI ≥ 2 years. However, in the group with TTI of 1–2 years, the elevated risk was observed only among those aged ≥60 years (p 30–32).

Cause-specific HRs were calculated to account for the competing risk of death, as shown in the appendix (p 33–35). In the results from the competing risk model, TTI of ≥1 year was an independent prognostic factor in the 30–39-year age group.

## Discussion

This study evaluated the impact of the TTI of antihypertensive medication for primary prevention on prognosis in patients diagnosed with hypertension at health examinations. The findings indicated that a delay in treatment initiation of ≥1 year may increase the risk of composite cerebro-cardiovascular events in patients aged ≥40 years. Furthermore, regarding the mortality risk, a delay in treatment initiation of ≥2 years was associated with an increased risk starting from the 40 s and highlights the importance of early therapeutic intervention.

The AHA/ACC guidelines recommend considering pharmacological therapy for high-risk cardiovascular patients or adults ≥65 years with SBP ≥ 130 mm Hg [11]. A recent AHA statement added that pharmacological therapy should also be considered in adults aged <65 years and in low-risk patients who remain uncontrolled after 3 to 6 months of lifestyle therapy [34]. The 2024 ESC guidelines recommend considering pharmacotherapy if BP exceeds 130/80 mm Hg after 3 months, but the use of the Systematic Coronary Risk Evaluation 2 (SCORE2) model has not been completely validated in people aged <40 years [12]. The Japanese hypertension guidelines recommend lifestyle therapy, followed by antihypertensive medication as needed, for high-risk patients with elevated BP (130–139/80–89 mm Hg) or hypertension ( ≥ 140/90 mm Hg) [8]. However, evidence on the TTI for patients with low-or-intermediate risk remains limited. Previous meta-analyses have confirmed the reduction in the relative risk of cerebrovascular disease with antihypertensive treatment in patients with low and intermediate risk [35–37]. However, results at the specific age of initiation of pharmacotherapy are limited, thus findings in our study at the TTI are novel. This study highlights the risks associated with delaying initiation of pharmacological therapy by ≥1 year, even in relatively low-risk patients ≥40 years, suggesting the potential benefit of early initiation of antihypertensive treatment. Moreover, multiple imputation and competing risk models may indicate an increased risk of duration of TTI from the 30 s.

The following hypotheses may explain how a prolonged TTI could impact prognosis. First, the progression of vascular damage due to prolonged periods of inadequate BP control may be a contributing factor. In the Cox proportional hazards model, individuals with a TTI of ≥2 years had a higher risk than those with a TTI of 1–2 years across all age groups aged ≥40 years. This suggests that a longer period of untreated time may lead to a sustained BP load, resulting in endothelial dysfunction, atherosclerosis, and left ventricular hypertrophy [38–40]. These pathophysiological changes are known to increase the risk of major cardiovascular events, such as stroke and myocardial infarction. In particular, the progression of atherosclerosis is more pronounced in middle-aged and older people, and early initiation of antihypertensive therapy to control BP may reduce the progression. Second, the loss of opportunity for organ-protective effects may be associated. Antihypertensive agents, such as angiotensin-converting enzyme (ACE) inhibitors and angiotensin II receptor blockers (ARBs), not only lower BP but also have renal and cardioprotective effects [41, 42]. Early use of these drugs in hypertensive patients may have prevented the progression of cardiovascular and renal disease in some populations. Thus, some may have lost the opportunity to benefit by delaying their use. Third, inadequate follow-up after a health check-up may result in other cardiovascular risk factors not being controlled. Delays in initiating antihypertensive therapy may reflect inadequate control of risk factors other than BP, such as diabetes, dyslipidemia, and smoking. These risk factors may progress without appropriate management, potentially leading to deteriorated outcomes [43].

To support these hypotheses, it is important to evaluate the impact of non-pharmacological therapy, such as lifestyle modifications (e.g., diet and exercise therapy). However, this study focused specifically on primary prevention by analyzing data from patients without prior cardiovascular or renal events. Therefore, the clinical significance of this study is that it raises an important consideration regarding the TTI in patients with de novo hypertension identified at health examinations.

### Limitations

This study has some potential limitations. First, in an observational study using real-world data, causality cannot be definitively established. It is difficult to fully adjust for confounding factors, such as socioeconomic conditions and patient behavioral characteristics, which may underlie delays in treatment initiation. Second, due to the nature of the database, detailed information on the types and dosages of antihypertensive medications and the effects of lifestyle modifications could not be considered, which requires cautious interpretation. Although our study did not adjust for social determinants of health or lifestyle factors such as diet, physical activity, or engagement in non-pharmacological treatment, these unmeasured confounders may have biased our results toward an underestimation of the true effect. For example, patients who delay treatment initiation may also adopt healthy behaviors that reduce cardiovascular risk. Therefore, the true effect of delayed treatment may be greater than what was observed in this study. Furthermore, mortality data are available only on a monthly basis; therefore, the analysis was conducted using mortality data obtained from the disease outcome with date information. On the other hand, previous studies reported that mortality data based on claims data was somewhat less sensitive [44]. Accordingly, some reliability concerns remain for the mortality information. Third, the database is limited to data from Japanese individuals or long-term foreign residents in Japan. Therefore, the external validity of the results may be limited when considering differences in healthcare systems and racial diversity in other countries. Fourth, as the aim of the study was to evaluate the efficacy of antihypertensive agents for primary prevention, patients who had an event, including death, before the initiation of antihypertensive drugs were excluded. Therefore, event rates may have been underestimated owing to survival bias. Additionally, for events that occur relatively early after drug initiation, the time relation between drug initiation and outcome may not be clearly established, which may partly explain the findings of the sensitivity analysis excluding early events. Fifth, we were unable to assess medication adherence after initiation of antihypertensive therapy due to data limitations. Adherence may mediate the association between delayed treatment initiation and cardiovascular outcomes. Sixth, the ages used for inclusion and exclusion criteria and for stratified analysis are based on age at physical examination. Therefore, residual confounding by age at initiation of antihypertensive medication may slightly affect the results. Finally, the study was based on data from health examinations and receipts, and the analysis relied on information recorded for reimbursement, which may introduce bias regarding the reflection of actual clinical conditions. Despite these limitations, this study provides new evidence on the TTI and prognosis in patients with hypertension identified by screening. It provides important insights that may contribute to the improvement of clinical guidelines, real-world practice, and public health policy. The accumulation of further evidence through interventional studies and international comparative research is expected in the future.

In conclusion, TTI ≥ 1 year was associated with an increased risk of composite cerebro-cardiovascular-renal events in patients aged ≥40 years. Furthermore, TTI ≥ 2 years was associated with a higher risk of mortality starting in the 40 s, highlighting the importance of early therapeutic intervention.

## Supplementary information

Supplementary information
