# Supplementary material for: Prognostic impact of the timing of antihypertensive medication initiation for hypertension detected at health screening on primary prevention of adverse cardiovascular events: Age-stratified real-world data analysis
Source: Hypertens Res. 2025 Jun 19;48(9):2401–12. doi: 10.1038/s41440-025-02249-1 (PMC12411247; doi:10.1038/s41440-025-02249-1)
Supplement: Supplementary file 1 — Supplementary information [file 41440_2025_2249_MOESM1_ESM.pdf]

Appendix

Table of contents

Supplementary Table 1. Blood pressure measurement protocol…………………………………………………………………..2

Supplementary Table 2. List of ATC codes used to identify antihypertensive agents……………………………………….3

Supplementary Figure 1. The correlation coefficients for each covariate……………………………………………………..4

Supplementary Table 3. Clinical characteristics of study population stratified by age group……………………………....5-11

Supplementary Figure 2. Sankey diagram visualizing hypertensive pharmacotherapy sequences……………………………12

Supplementary Table 4. Cumulative incidence rates for the events included in the primary endpoint……….……………..13

Supplementary Figure 3. Kaplan-Meier curves for the primary outcome for each TTI group stratified by age………..14

Supplementary Table 5. Sensitivity analysis: multivariate Cox proportional analysis in the Elevated blood pressure (130–139 mm Hg systolic and/or 80-89 mm Hg diastolic) cohort………………...……………………………………………….15-17

Supplementary Table 6. Sensitivity analysis: multivariate Cox proportional analysis in the Grade I or higher hypertension (≥140 mm Hg systolic and/or ≥90 mm Hg diastolic) cohort……………………………………………………….…...18-20

Supplementary Table 7. Sensitivity analysis: multivariate Cox proportional analysis in a cohort of participants with hypertension detected at a second or higher physical examination……………………………………………………….….21-23

Supplementary Table 8. Sensitivity analysis: multivariate Cox proportional analysis performed by changing the covariate from visceral obesity (waist circumference) to overweight (body mass index)……………………………………………….24-26

Supplementary Table 9. Sensitivity analysis: multivariate Cox proportional analysis after multiple imputation for missing data………………………………………………………………………….……...……………...…………………27-29

Supplementary Table 10. Sensitivity analysis: multivariate Cox proportional analysis excluding events that occurred within the first year after treatment initiation…………………………………….……...……………...…………………30-32

Supplementary Table 11. Sensitivity analysis: Hazard ratios for the primary endpoints considering competing risks of death using Fine-Gray modelling.……………………………………………………………………………...………..….33-35

Supplementary Table 1. Blood pressure measurement protocol

Category	Protocol
1. Device	Blood pressure measurements should be taken with either a mercury or aneroid sphygmomanometer whose accuracy has been validated. Alternatively, a validated electronic sphygmomanometer may be used.<br>For auscultation, a cuff with a bladder 13 cm wide and 22-24 cm long is recommended. For individuals with arm circumferences less than 27 cm, a pediatric cuff should be selected. Conversely, for individuals with larger arms (arm circumference ≥34 cm), a large adult cuff is recommended.
2. Measurement conditions	Measurements should be taken in a quiet environment at a comfortable room temperature. The subject should sit quietly for several minutes in a chair with back support, feet flat on the floor and legs uncrossed. Conversation should be avoided during measurement. In addition, smoking and the consumption of alcohol or caffeine should be avoided prior to measurement.
3. Measurement methods	The forearm should rest comfortably on a table and the cuff should be applied so that the bottom of the cuff is 2-3 cm above the elbow crease. Ensure that the cuff is at heart level, approximately at the midpoint of the sternum or the fourth intercostal space. When using the auscultatory method, rapidly inflate the cuff while palpating the radial or brachial artery until the pulse disappears, then inflate an additional 30 mmHg. Deflate the cuff gradually at a rate of 2-3 mmHg per heartbeat or per second. Systolic blood pressure is determined by noting the pressure at which the first Korotkoff sound occurs and diastolic blood pressure by the disappearance of the fifth Korotkoff sound.
4. Frequency of measurement	Blood pressure should be measured at least twice, with an interval of 1-2 minutes between measurements. If the two readings differ significantly (a difference of approximately 5 mmHg or more), additional measurements should be taken to ensure accuracy.

From Table 2-1 of the Japanese Society of Hypertension Guidelines for the Management of Hypertension (JSH 2019) [Uemura S, et al. Hypertens Res. 2019;42:1235-481.]

Supplementary Table 2. List of ATC codes used to identify antihypertensive agents.

Antihypertensive agents	Variable name	ATC codes
Calcium channel blockers	CCB	C08CA, C08CX, C08DB, C08GA, C09BB, C09DB, C09DX01, C09DX03, C09DX08, C10BX03
Beta blockers	BETABLOCK	C07AA03, C07AA05, C07AA12, C07AA15, C07AB02, C07AB03, C07AB05, C07AB06, C07AB07, C07AB08, C07AG01, C07AG02
Angiotensin-converting enzyme inhibitors	ACEI	C09AA, C09BA, C09BB, C09BX
Angiotensin II receptor blockers	ARB	C09CA, C09DA, C09DB, C09DX01, C09DX02, C09DX03, C09DX05, C09DX06, C09DX07, C09DX08
Direct renin inhibitors	DRI	C09XA02
Angiotensin-receptor neprilysin inhibitor	ARNI	C09DX04
Antihypertensive diuretics	TZD	C03AA03, C03AA06, C03BA05, C03BA11, C09DA, C09DX08
Alpha-adrenergic agents	ARA	C02AB01, C02AC01
Alpha blockers	ALPHABLOCK	C02CA01, C02CA04, C02CA06

Antihypertensive drugs approved and marketed primarily in Japan were identified using anatomical therapeutic chemical Classification (ATC) codes. Fixed-dose combinations were processed to identify both or multi-types. The ATC codes used can be found in the ATC/DDD Index 2024 published by the WHO Collaborating Centre for Drug Statistics Methodology (https://atcddd.fhi.no/atc_ddd_index/).

Supplementary Figure 1. Correlation coefficients for each covariate.

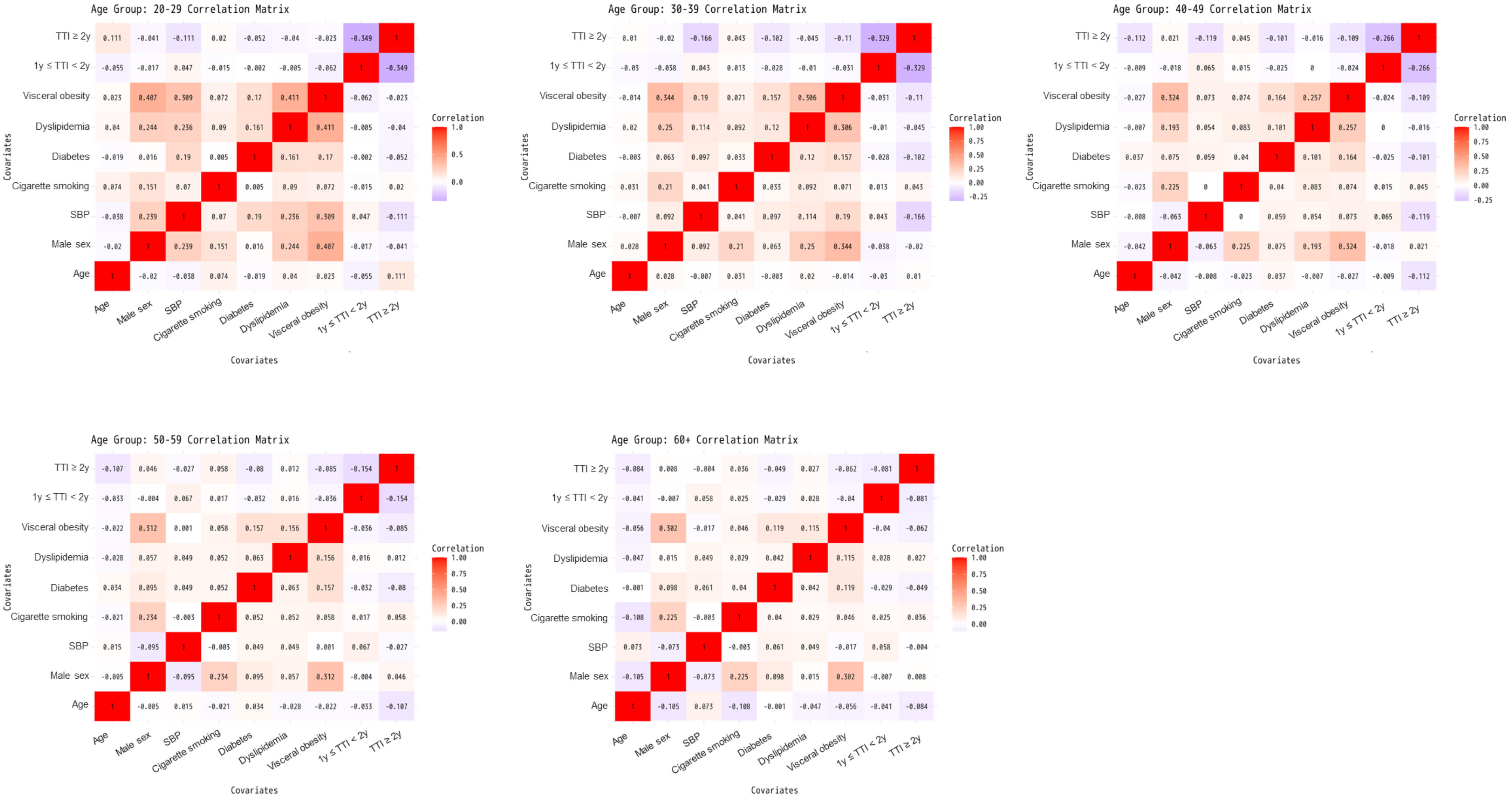

SBP: systolic blood pressure; TTI: time to treatment initiation.

Supplementary Table 3. Age-group stratified clinical characteristics of the study population

		Time to treatment initiation (TTI)				
**Variable**	**Missing**	**< 1 year**	**1-2 years**	**≥ 2 years**	***P*-value**	**Std diff**
N						
20-29 years		1402	559	1452		
30-39 years		11847	3776	11384		
40-49 years		80490	17518	53184		
50-59 years		150353	19110	40765		
≥60 years		113735	8547	11887		
Age, years						
20-29 years	0	25.98 (2.44)	25.92 (2.56)	26.18 (2.49)	0.049	0.064
30-39 years	0	36.49 (2.58)	36.24 (2.68)	36.41 (2.57)	<0.001	0.065
40-49 years	0	45.31 (2.84)	44.93 (2.88)	44.56 (2.90)	<0.001	0.174
50-59 years	0	54.67 (2.84)	54.18 (2.82)	53.84 (2.78)	<0.001	0.198
≥60 years	0	64.17 (3.64)	63.46 (3.24)	63.03 (2.91)	<0.001	0.229
Male sex, n (%)						
20-29 years	0	1105 (78.82)	420 (75.13)	1147 (78.99)	0.140	0.060
30-39 years	0	9218 (77.81)	2740 (72.56)	8809 (77.38)	<0.001	0.086
40-49 years	0	57943 (71.99)	12327 (70.37)	39677 (74.60)	<0.001	0.063
50-59 years	0	103441 (68.80)	13287 (69.53)	30495 (74.81)	<0.001	0.085
≥60 years	0	75072 (66.01)	5568 (65.15)	8102 (68.16)	<0.001	0.041
Body mass index, kg/m^2						
20-29 years	1	27.72 (6.79)	27.18 (6.79)	26.53 (5.94)	<0.001	0.123
30-39 years	13	28.18 (5.99)	26.73 (5.34)	26.03 (4.90)	<0.001	0.267
40-49 years	71	26.85 (4.98)	25.75 (4.48)	25.11 (4.05)	<0.001	0.257
50-59 years	89	25.43 (4.14)	24.47 (3.66)	24.11 (3.42)	<0.001	0.233
≥60 years	54	24.42 (3.49)	23.59 (3.21)	23.38 (3.11)	<0.001	0.210
Overweight, n (%)						
20-29 years	1	826 (58.92)	307 (54.92)	767 (52.82)	0.014	0.093

30-39 years	13	8003 (67.55)	2241 (59.35)	6081 (53.42)	<0.001	0.198
40-49 years	71	826 (58.92)	307 (54.92)	767 (52.82)	<0.001	0.201
50-59 years	89	75086 (49.94)	7648 (40.02)	14449 (35.44)	<0.001	0.199
≥60 years	54	45198 (39.74)	2574 (30.12)	3291 (27.69)	<0.001	0.172
Waist circumference, cm						
20-29 years	1522	92.18 (17.67)	90.28 (17.57)	89.28 (15.43)	0.003	0.117
30-39 years	3690	93.44 (14.28)	89.92 (13.17)	88.41 (12.01)	<0.001	0.258
40-49 years	1632	90.73 (12.18)	88.07 (11.14)	86.67 (10.23)	<0.001	0.243
50-59 years	1694	88.40 (10.44)	86.01 (9.50)	85.17 (9.02)	<0.001	0.221
≥60 years	693	86.73 (9.16)	84.59 (8.76)	84.11 (8.48)	<0.001	0.198
Visceral obesity, n (%)						
20-29 years	1522	536 (38.23)	191 (34.17)	360 (24.79)	<0.001	0.313
30-39 years	3690	7315 (61.75)	1935 (51.24)	5128 (45.05)	<0.001	0.250
40-49 years	1632	536 (38.23)	191 (34.17)	360 (24.79)	<0.001	0.179
50-59 years	1694	83739 (55.69)	9014 (47.17)	17988 (44.13)	<0.001	0.162
≥60 years	693	56983 (50.10)	3496 (40.90)	4570 (38.45)	<0.001	0.160
Systolic blood pressure, mm Hg						
20-29 years	0	140.63 (16.30)	140.94 (15.26)	137.11 (12.36)	<0.001	0.172
30-39 years	0	141.51 (16.65)	141.08 (15.26)	136.27 (12.76)	<0.001	0.242
40-49 years	0	139.05 (15.90)	140.81 (15.36)	135.66 (12.76)	<0.001	0.238
50-59 years	0	137.88 (14.81)	141.32 (15.35)	137.30 (13.53)	<0.001	0.181
≥60 years	0	140.02 (14.19)	143.55 (15.34)	140.17 (13.92)	<0.001	0.160
Diastolic blood pressure, mm Hg						
20-29 years	0	87.07 (12.85)	86.90 (11.49)	83.83 (9.55)	<0.001	0.203
30-39 years	0	91.30 (11.96)	91.60 (10.81)	87.47 (9.05)	<0.001	0.271
40-49 years	0	90.05 (10.47)	91.37 (10.25)	87.90 (8.61)	<0.001	0.242
50-59 years	0	87.42 (9.14)	89.82 (9.77)	87.54 (8.55)	<0.001	0.174
≥60 years	0	83.98 (9.21)	86.47 (9.83)	85.37 (8.83)	<0.001	0.178
Stage 1 hypertension, n (%)						
20-29 years	0	690 (49.22)	285 (50.98)	893 (61.50)	<0.001	0.179

30-39 years	0	5027 (42.43)	1472 (38.98)	6392 (56.15)	<0.001	0.236
40-49 years	0	38003 (47.21)	6840 (39.05)	29516 (55.50)	<0.001	0.224
50-59 years	0	79196 (52.67)	7744 (40.52)	21254 (52.14)	<0.001	0.167
≥60 years	0	59079 (51.94)	3434 (40.18)	5831 (49.05)	<0.001	0.158
Stage 2 hypertension, n (%)						
20-29 years	0	712 (50.78)	274 (49.02)	559 (38.50)	<0.001	0.179
30-39 years	0	6820 (57.57)	2304 (61.02)	4992 (43.85)	<0.001	0.236
40-49 years	0	42487 (52.79)	10678 (60.95)	23668 (44.50)	<0.001	0.224
50-59 years	0	71157 (47.33)	11366 (59.48)	19511 (47.86)	<0.001	0.167
≥60 years	0	54656 (48.06)	5113 (59.82)	6056 (50.95)	<0.001	0.158
Triglycerides, mg/dL						
20-29 years	628	106 (70-162)	101 (68-169)	103 (66-160)	0.104	0.059
30-39 years	901	125 (83-190)	116 (75-175)	114 (76-174)	<0.001	0.064
40-49 years	339	120 (81-179)	113 (76-170)	111 (75-167)	<0.001	0.057
50-59 years	368	113 (79-166)	108 (75-159)	107 (75-158)	<0.001	0.039
≥60 years	268	107 (77-152)	102 (73-146)	102 (74-147)	<0.001	0.031
HDL cholesterol, mg/dL						
20-29 years	624	54.75 (15.07)	55.88 (15.94)	55.53 (14.50)	0.295	0.053
30-39 years	878	54.63 (14.87)	56.19 (14.93)	56.13 (15.17)	<0.001	0.074
40-49 years	254	57.76 (16.07)	58.97 (16.24)	58.74 (15.89)	<0.001	0.051
50-59 years	301	61.19 (16.94)	62.32 (17.45)	61.74 (16.79)	<0.001	0.046
≥60 years	249	62.30 (16.76)	63.62 (17.21)	63.08 (16.76)	<0.001	0.054
LDL cholesterol, mg/dL						
20-29 years	648	119.37 (34.62)	120.14 (33.36)	117.58 (33.46)	0.278	0.063
30-39 years	960	125.34 (32.56)	126.98 (33.79)	125.66 (32.97)	0.037	0.032
40-49 years	361	124.77 (31.35)	128.42 (32.96)	127.76 (31.96)	<0.001	0.075
50-59 years	421	123.96 (30.53)	130.47 (32.64)	129.64 (31.79)	<0.001	0.139
≥60 years	282	121.49 (29.48)	129.85 (31.85)	129.27 (31.18)	<0.001	0.180
AST, U/L						
20-29 years	572	30.66 (20.58)	30.16 (23.12)	28.33 (18.87)	0.013	0.064

30-39 years	841	29.86 (20.28)	27.48 (17.63)	26.49 (15.99)	<0.001	0.122
40-49 years	238	27.40 (16.93)	25.63 (15.46)	24.55 (12.98)	<0.001	0.125
50-59 years	286	26.45 (14.80)	25.02 (13.45)	24.40 (12.38)	<0.001	0.099
≥60 years	237	25.66 (13.33)	24.57 (11.32)	24.37 (10.99)	<0.001	0.070
ALT, U/L						
20-29 years	572	48.74 (44.87)	47.63 (53.88)	44.21 (45.21)	0.046	0.054
30-39 years	841	43.70 (36.88)	38.35 (32.83)	37.00 (31.91)	<0.001	0.131
40-49 years	236	35.11 (27.04)	31.39 (24.86)	30.13 (23.07)	<0.001	0.132
50-59 years	282	29.53 (21.05)	26.85 (19.45)	25.98 (18.22)	<0.001	0.119
≥60 years	226	25.34 (17.39)	23.18 (14.60)	23.08 (14.64)	<0.001	0.094
γ-glutamyl transpeptidase, IU/L						
20-29 years	597	33 (20-62)	32 (20-57)	30 (19-54)	0.072	0.055
30-39 years	866	41 (25-72)	37 (22-64)	35 (22-61)	<0.001	0.106
40-49 years	308	39 (24-69)	36 (22-63)	35 (22-60)	<0.001	0.076
50-59 years	404	38 (24-66)	35 (22-60)	35 (22-59)	<0.001	0.063
≥60 years	282	32 (21-54)	30 (20-51)	30 (20-50)	<0.001	0.043
Fasting blood glucose, mg/dL						
20-29 years	1436	91 (85-98)	90 (84-97.5)	90 (84-96)	0.009	0.095
30-39 years	5747	95 (88-104)	93 (87-100)	92 (86-99)	<0.001	0.178
40-49 years	22490	95 (88-104)	93 (88-100)	92 (86-99)	<0.001	0.184
50-59 years	5747	95 (88-104)	93 (87-100)	92 (86-99)	<0.001	0.169
≥60 years	18916	102 (94-114)	99 (92-109)	99 (92-108)	<0.001	0.140
HbA_{1c}, %						
20-29 years	1349	5.56 (1.00)	5.51 (0.91)	5.41 (0.74)	0.001	0.122
30-39 years	4865	5.75 (1.05)	5.58 (0.96)	5.52 (0.78)	<0.001	0.170
40-49 years	16399	5.80 (0.97)	5.66 (0.90)	5.58 (0.75)	<0.001	0.169
50-59 years	21596	5.87 (0.87)	5.75 (0.86)	5.67 (0.72)	<0.001	0.155
≥60 years	16208	5.91 (0.76)	5.82 (0.77)	5.77 (0.69)	<0.001	0.134
Hemoglobin, g/dL						
20-29 years	857	15.35 (1.45)	15.37 (1.43)	15.21 (1.43)	0.040	0.080

30-39 years	6031	15.15 (1.59)	15.05 (1.61)	15.08 (1.53)	0.003	0.045
40-49 years	43251	14.85 (1.62)	14.84 (1.70)	14.81 (1.66)	<0.001	0.020
50-59 years	62213	14.74 (1.35)	14.78 (1.44)	14.80 (1.38)	<0.001	0.028
≥60 years	41995	14.47 (1.30)	14.54 (1.29)	14.54 (1.26)	<0.001	0.038
Cigarette smoking, n (%)						
20-29 years	27	341 (24.32)	135 (24.15)	452 (31.13)	<0.001	0.147
30-39 years	79	3623 (30.58)	1304 (34.53)	4253 (37.36)	<0.001	0.096
40-49 years	146	23581 (29.30)	5832 (33.29)	18441 (34.67)	<0.001	0.075
50-59 years	27	38993 (25.93)	5795 (30.32)	13527 (33.18)	<0.001	0.104
≥60 years	27	22338 (19.64)	2124 (24.85)	3041 (25.58)	<0.001	0.091
Diabetes, n (%)						
20-29 years	1437	53 (3.78)	13 (2.33)	27 (1.86)	<0.001	0.133
30-39 years	5750	942 (7.95)	159 (4.21)	316 (2.78)	<0.001	0.164
40-49 years	22526	7695 (9.56)	961 (5.49)	2038 (3.83)	<0.001	0.157
50-59 years	1437	17414 (11.58)	1385 (7.25)	2221 (5.45)	<0.001	0.150
≥60 years	1437	14285 (12.56)	719 (8.41)	846 (7.12)	<0.001	0.121
Dyslipidemia, n (%)						
20-29 years	666	543 (38.73)	213 (38.10)	479 (32.99)	<0.001	0.108
30-39 years	1003	6528 (55.10)	1932 (51.17)	5725 (50.29)	<0.001	0.083
40-49 years	515	43425 (53.95)	9378 (53.53)	27856 (52.38)	<0.001	0.023
50-59 years	666	76568 (50.93)	10330 (54.06)	21576 (52.93)	<0.001	0.041
≥60 years	666	51376 (45.17)	4372 (51.15)	5985 (50.35)	<0.001	0.083
Antihypertensive agents, n (%)						
Calcium channel blockers						
20-29 years	0	820 (58.49)	371 (66.37)	943 (64.94)	<0.001	0.096
30-39 years	0	9026 (76.19)	2823 (74.76)	8557 (75.17)	0.091	0.021
40-49 years	0	820 (58.49)	371 (66.37)	943 (64.94)	<0.001	0.042
50-59 years	0	118725 (78.96)	14557 (76.17)	30573 (75.00)	<0.001	0.055
≥60 years	0	89192 (78.42)	6348 (74.27)	8860 (74.54)	<0.001	0.062
Beta blockers						

20-29 years	0	385 (27.46)	137 (24.51)	305 (21.01)	<0.001	0.095
30-39 years	0	2240 (18.91)	695 (18.41)	1695 (14.89)	<0.001	0.082
40-49 years	0	385 (27.46)	137 (24.51)	305 (21.01)	<0.001	0.090
50-59 years	0	22915 (15.24)	2853 (14.93)	5145 (12.62)	<0.001	0.070
≥60 years	0	15601 (13.72)	1095 (12.81)	1501 (12.63)	<0.001	0.051
Angiotensin-converting enzyme inhibitors						
20-29 years	0	87 (6.21)	20 (3.58)	55 (3.79)	0.004	0.080
30-39 years	0	638 (5.39)	217 (5.75)	560 (4.92)	0.090	0.025
40-49 years	0	87 (6.21)	20 (3.58)	55 (3.79)	<0.001	0.033
50-59 years	0	7651 (5.09)	943 (4.93)	1749 (4.29)	<0.001	0.034
≥60 years	0	6160 (5.42)	422 (4.94)	531 (4.47)	<0.001	0.046
Angiotensin II receptor blockers						
20-29 years	0	716 (51.07)	218 (39.00)	703 (48.42)	<0.001	0.164
30-39 years	0	8123 (68.57)	2134 (56.51)	6735 (59.16)	<0.001	0.167
40-49 years	0	716 (51.07)	218 (39.00)	703 (48.42)	<0.001	0.033
50-59 years	0	107358 (71.40)	11634 (60.88)	23480 (57.60)	<0.001	0.189
≥60 years	0	76283 (67.07)	4670 (54.64)	6260 (52.66)	<0.001	0.191
Direct renin inhibitors						
20-29 years	0	5 (0.36)	1 (0.18)	2 (0.14)	0.461	0.055
30-39 years	0	20 (0.17)	2 (0.05)	12 (0.11)	0.156	0.024
40-49 years	0	5 (0.36)	1 (0.18)	2 (0.14)	<0.001	0.165
50-59 years	0	259 (0.17)	21 (0.11)	16 (0.04)	<0.001	0.028
≥60 years	0	180 (0.16)	8 (0.09)	3 (0.03)	0.001	0.030
Angiotensin receptor neprilysin inhibitor						
20-29 years	0	0 (0.00)	0 (0.00)	1 (0.07)	0.509	<0.001
30-39 years	0	4 (0.03)	0 (0.00)	1 (0.01)	0.250	0.019
40-49 years	0	0 (0.00)	0 (0.00)	1 (0.07)	0.428	0.005
50-59 years	0	18 (0.01)	6 (0.03)	6 (0.01)	0.106	0.003
≥60 years	0	8 (0.01)	1 (0.01)	2 (0.02)	0.498	0.004
Antihypertensive diuretics						

20-29 years	0	104 (7.42)	29 (5.19)	87 (5.99)	0.125	0.063
30-39 years	0	1331 (11.23)	311 (8.24)	898 (7.89)	<0.001	0.074
40-49 years	0	104 (7.42)	29 (5.19)	87 (5.99)	<0.001	0.132
50-59 years	0	17913 (11.91)	1580 (8.27)	2523 (6.19)	<0.001	0.133
≥60 years	0	11558 (10.16)	552 (6.46)	591 (4.97)	<0.001	0.131
Alpha-adrenergic agents						
20-29 years	0	8 (0.57)	1 (0.18)	3 (0.21)	0.195	0.038
30-39 years	0	10 (0.08)	2 (0.05)	6 (0.05)	0.607	0.008
40-49 years	0	8 (0.57)	1 (0.18)	3 (0.21)	0.032	0.009
50-59 years	0	48 (0.03)	12 (0.06)	12 (0.03)	0.080	0.010
≥60 years	0	17 (0.01)	1 (0.01)	1 (0.01)	0.834	0.004
Alpha blockers						
20-29 years	0	41 (2.92)	23 (4.11)	55 (3.79)	0.307	0.043
30-39 years	0	537 (4.53)	141 (3.73)	316 (2.78)	<0.001	0.061
40-49 years	0	41 (2.92)	23 (4.11)	55 (3.79)	<0.001	0.068
50-59 years	0	6073 (4.04)	608 (3.18)	1057 (2.59)	<0.001	0.054
≥60 years	0	4249 (3.74)	263 (3.08)	294 (2.47)	<0.001	0.048

Categorical variables are expressed as frequency (proportion) unless otherwise indicated. Continuous variables with normal distributions are shown as mean (standard deviation), and medians (interquartile ranges) were shown for the data with skewed distribution.

Supplementary Figure 2. Sankey diagram depicting sequences of hypertensive pharmacotherapy

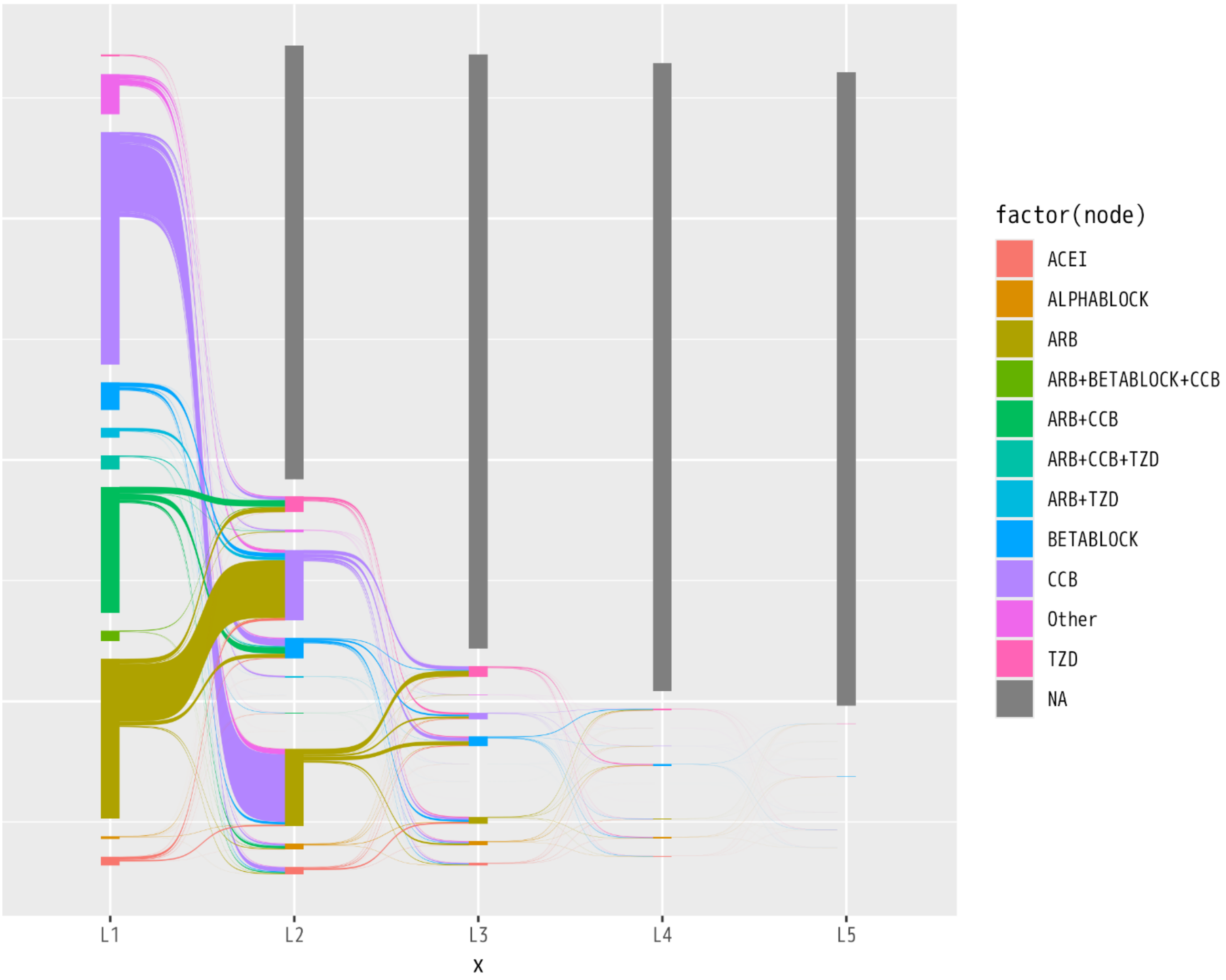

Sankey diagrams depict sequences of hypertensive pharmacotherapy to illustrate a flow from one set of values to another. These diagrams do not reflect the treatment duration or the timing of switching treatment. The abbreviations are as defined in Supplementary Table 2. ALPHABLOCK, alpha blockers; ACEI, Angiotensin-converting enzyme inhibitors; ARA, alpha-adrenergic agents; ARB, angiotensin II receptor blockers; ARNI, angiotensin-receptor neprilysin inhibitor; BETABLOCK, beta blockers CCB, calcium channel blockers; DRI, direct renin inhibitors; TZD, antihypertensive diuretics

Supplementary Table 4. Cumulative event rates for the events included in the primary endpoint.

Cumulative event rates, % (95% CI)	TTI group	Follow-up, years				
		1	2	3	4	5
Cardiovascular death	<1 year	0.14 (0.13-0.15)	0.24 (0.22-0.25)	0.34 (0.31-0.36)	0.42 (0.39-0.45)	0.51 (0.48-0.55)
	1-2 years	0.33 (0.27-0.38)	0.46 (0.40-0.53)	0.55 (0.47-0.63)	0.65 (0.56-0.75)	0.72 (0.61-0.83)
	≥2 years	0.37 (0.33-0.40)	0.48 (0.44-0.53)	0.57 (0.51-0.63)	0.65 (0.59-0.72)	0.77 (0.69-0.86)
Acute coronary syndrome	<1 year	0.21 (0.19-0.23)	0.35 (0.33-0.37)	0.52 (0.49-0.55)	0.67 (0.63-0.70)	0.84 (0.80-0.89)
	1-2 years	0.46 (0.40-0.52)	0.66 (0.58-0.74)	0.80 (0.70-0.90)	0.95 (0.83-1.06)	1.08 (0.94-1.21)
	≥2 years	0.53 (0.48-0.57)	0.71 (0.65-0.77)	0.86 (0.79-0.94)	1.00 (0.92-1.09)	1.18 (1.07-1.29)
Heart failure	<1 year	0.18 (0.17-0.20)	0.31 (0.29-0.33)	0.45 (0.42-0.48)	0.57 (0.54-0.60)	0.71 (0.67-0.75)
	1-2 years	0.41 (0.35-0.47)	0.57 (0.50-0.65)	0.70 (0.61-0.79)	0.81 (0.70-0.91)	0.92 (0.79-1.04)
	≥2 years	0.46 (0.42-0.50)	0.61 (0.56-0.66)	0.73 (0.67-0.79)	0.84 (0.76-0.91)	1.00 (0.90-1.10)
Stoke	<1 year	0.19 (0.17-0.20)	0.32 (0.30-0.35)	0.48 (0.45-0.51)	0.62 (0.59-0.66)	0.77 (0.73-0.82)
	1-2 years	0.41 (0.35-0.47)	0.58 (0.51-0.66)	0.71 (0.62-0.80)	0.86 (0.75-0.97)	1.00 (0.86-1.13)
	≥2 years	0.46 (0.42-0.50)	0.63 (0.58-0.69)	0.77 (0.70-0.83)	0.89 (0.81-0.97)	1.05 (0.95-1.16)
Primary outcome (composite cerebro-cardiovascular events)	<1 year	0.61 (0.52-0.70)	0.89 (0.78-1.01)	1.13 (0.99-1.28)	1.37 (1.20-1.54)	1.47 (1.29-1.65)
	1-2 years	1.13 (0.83-1.43)	1.46 (1.09-1.84)	1.73 (1.29-2.17)	1.81 (1.34-2.28)	2.00 (1.46-2.54)
	≥2 years	1.29 (1.07-1.50)	1.65 (1.36-1.94)	1.94 (1.59-2.30)	2.20 (1.78-2.62)	2.20 (1.78-2.62)
Secondary outcome (all-cause death)	<1 year	0.22 (0.20-0.24)	0.37 (0.35-0.39)	0.55 (0.52-0.58)	0.71 (0.67-0.75)	0.90 (0.85-0.95)
	1-2 years	0.48 (0.41-0.54)	0.68 (0.60-0.76)	0.83 (0.73-0.93)	0.99 (0.87-1.11)	1.14 (1.00-1.28)
	≥2 years	0.54 (0.49-0.59)	0.73 (0.67-0.79)	0.89 (0.82-0.97)	1.04 (0.95-1.12)	1.22 (1.11-1.33)

TTI: time to treatment initiation.

Supplementary Figure 3. Kaplan-Meier curves for the primary outcome for each TTI group stratified by age.

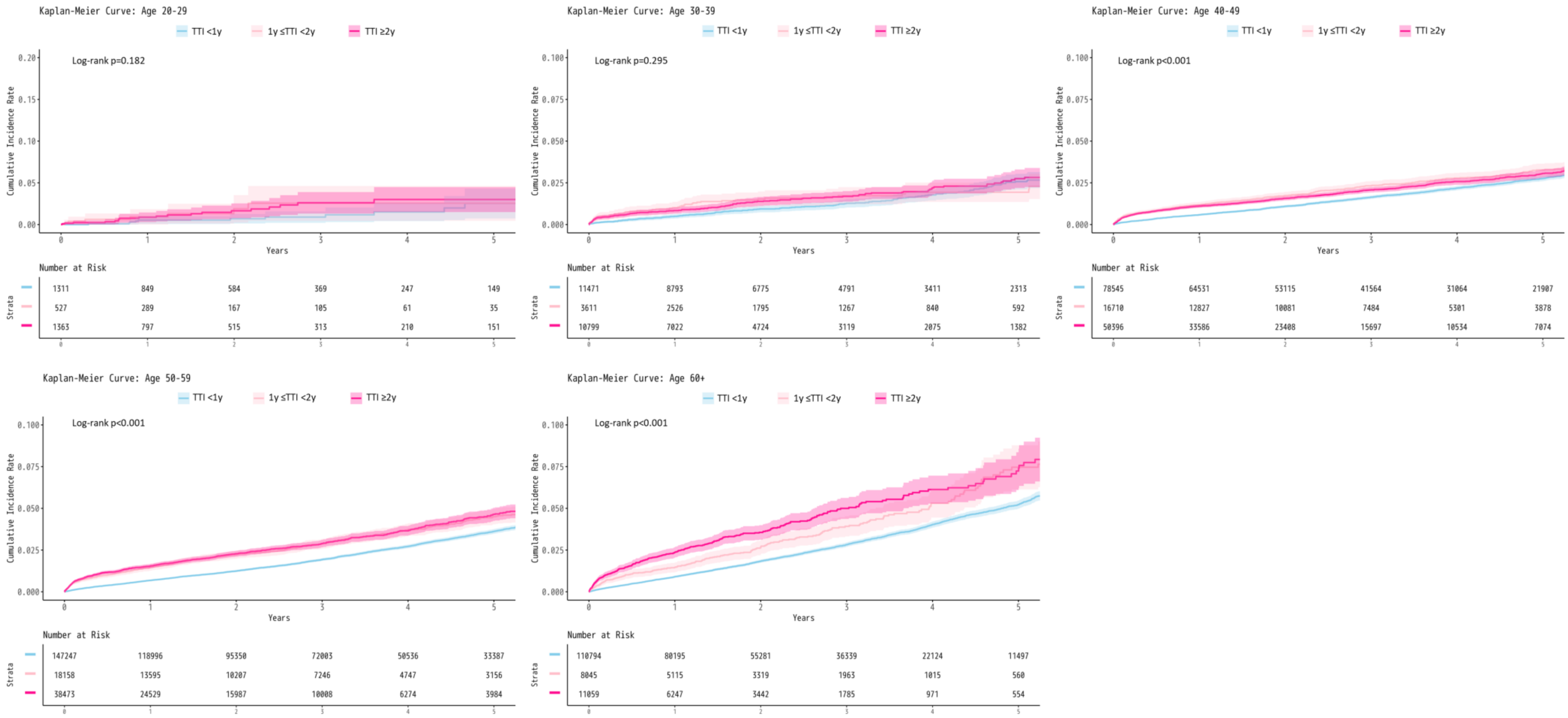

TTI: time to treatment initiation.

Supplementary Table 5. Sensitivity analysis: multivariate Cox proportional analysis in the Elevated blood pressure (130–139 mm Hg systolic and/or 80-89 mm Hg diastolic) cohort.

**Age group: 20–29 years**	**Univariate analysis**			**Multivariate analysis**		
**Variable**	**Crude HR**	**95% CI**	**p-value**	**Adjusted HR**	**95% CI**	**p-value**
1 year ≤ TTI < 2 years	4.246	0.382-47.20	0.239	1.136	0.064-20.08	0.931
TTI ≥ 2 years	4.421	0.493-39.61	0.184	0.171	0.005-6.264	0.337
Age	0.980	0.813-1.181	0.834	0.905	0.640-1.282	0.575
Male sex	1.692	0.485-5.902	0.409	0.205	0.026-1.629	0.134
Systolic blood pressure	0.987	0.924-1.054	0.695	0.946	0.835-1.071	0.380
Cigarette smoking	0.759	0.247-2.335	0.630	-	-	-
Dyslipidemia	3.056	0.960-9.726	0.059	3.068	0.437-21.56	0.260
Diabetes	-	-	-	-	-	-
Visceral obesity	1.738	0.387-7.810	0.471	4.585	0.516-40.74	0.172
**Age group: 30–39 years**	**Univariate analysis**			**Multivariate analysis**		
**Variable**	**Crude HR**	**95% CI**	**p-value**	**Adjusted HR**	**95% CI**	**p-value**
1 year ≤ TTI < 2 years	1.208	0.736-1.984	0.455	1.079	0.582-1.999	0.810
TTI ≥ 2 years	1.716	1.112-2.648	0.015	1.540	0.907-2.615	0.110
Age	0.999	0.945-1.056	0.971	0.995	0.922-1.073	0.891
Male sex	1.141	0.815-1.599	0.442	1.016	0.644-1.604	0.945
Systolic blood pressure	0.989	0.970-1.009	0.284	0.977	0.954-1.000	0.052
Cigarette smoking	1.196	0.890-1.606	0.235	1.135	0.770-1.671	0.522
Dyslipidemia	1.311	0.982-1.750	0.066	1.432	0.974-2.106	0.068
Diabetes	1.450	0.801-2.626	0.220	1.354	0.694-2.642	0.374
Visceral obesity	1.122	0.812-1.549	0.486	0.998	0.667-1.495	0.994
**Age group: 40–49 years**	**Univariate analysis**			**Multivariate analysis**		

Variable	Crude HR	95% CI	p-value	Adjusted HR	95% CI	p-value
1 year ≤ TTI < 2 years	1.480	1.249-1.754	<0.001	1.524	1.266-1.834	<0.001
TTI ≥ 2 years	1.387	1.204-1.598	<0.001	1.401	1.195-1.641	<0.001
Age	1.027	1.008-1.046	0.005	1.035	1.014-1.056	0.001
Male sex	1.189	1.045-1.352	0.009	1.133	0.970-1.323	0.116
Systolic blood pressure	1.005	0.997-1.013	0.201	1.002	0.994-1.011	0.555
Cigarette smoking	1.357	1.218-1.512	<0.001	1.341	1.186-1.516	<0.001
Dyslipidemia	1.136	1.022-1.262	0.018	1.113	0.987-1.255	0.082
Diabetes	1.514	1.267-1.809	<0.001	1.478	1.230-1.776	<0.001
Visceral obesity	1.101	0.990-1.226	0.077	1.025	0.902-1.164	0.709

**Age group: 50–59 years**	**Univariate analysis**			**Multivariate analysis**		
**Variable**	**Crude HR**	**95% CI**	**p-value**	**Adjusted HR**	**95% CI**	**p-value**
1 year ≤ TTI < 2 years	1.466	1.272-1.689	<0.001	1.536	1.317-1.792	<0.001
TTI ≥ 2 years	1.401	1.244-1.578	<0.001	1.458	1.279-1.661	<0.001
Age	1.043	1.028-1.058	<0.001	1.050	1.033-1.066	<0.001
Male sex	1.652	1.485-1.838	<0.001	1.463	1.291-1.657	<0.001
Systolic blood pressure	0.994	0.989-1.000	0.045	0.994	0.988-1.000	0.070
Cigarette smoking	1.357	1.247-1.476	<0.001	1.235	1.125-1.357	<0.001
Dyslipidemia	1.109	1.024-1.202	0.011	1.032	0.945-1.127	0.488
Diabetes	1.384	1.225-1.564	<0.001	1.286	1.134-1.458	<0.001
Visceral obesity	1.263	1.163-1.370	<0.001	1.183	1.077-1.300	<0.001

**Age group: ≥60 years**	**Univariate analysis**			**Multivariate analysis**		
**Variable**	**Crude HR**	**95% CI**	**p-value**	**Adjusted HR**	**95% CI**	**p-value**
1 year ≤ TTI < 2 years	1.166	0.936-1.453	0.171	1.107	0.866-1.414	0.417
TTI ≥ 2 years	1.451	1.217-1.729	<0.001	1.487	1.228-1.800	<0.001
Age	1.077	1.062-1.092	<0.001	1.088	1.071-1.105	<0.001

Male sex	1.351	1.207-1.513	<0.001	1.298	1.136-1.483	<0.001
Systolic blood pressure	1.002	0.994-1.009	0.680	1.000	0.991-1.008	0.939
Cigarette smoking	1.355	1.213-1.514	<0.001	1.365	1.206-1.546	<0.001
Dyslipidemia	1.123	1.018-1.237	0.020	1.108	0.996-1.232	0.059
Diabetes	1.117	0.962-1.297	0.145	1.047	0.899-1.218	0.557
Visceral obesity	1.250	1.133-1.379	<0.001	1.174	1.050-1.313	0.005

Multivariate Cox regression analysis for predictors of primary outcomes was conducted after adjustment by time to treatment initiation groups, age, male sex, systolic blood pressure, cigarette smoking, dyslipidemia, diabetes, and visceral obesity. HR: hazard ratio; CI: confidence index; TTI: time to treatment initiation.

Supplementary Table 6. Sensitivity analysis: multivariate Cox proportional analysis in the Grade I or higher hypertension (≥140 mm Hg systolic and/or ≥90 mm Hg diastolic) cohort

**Age group: 20–29 years**	**Univariate analysis**			**Multivariate analysis**		
**Variable**	**Crude HR**	**95% CI**	**p-value**	**Adjusted HR**	**95% CI**	**p-value**
1 year ≤ TTI < 2 years	2.006	0.656-6.138	0.222	1.020	0.090-11.61	0.987
TTI ≥ 2 years	1.261	0.421-3.778	0.679	1.952	0.265-14.38	0.511
Age	1.058	0.893-1.253	0.513	1.106	0.772-1.586	0.582
Male sex	0.846	0.290-2.464	0.759	0.428	0.058-3.147	0.404
Systolic blood pressure	1.034	1.008-1.060	0.010	1.009	0.951-1.070	0.766
Cigarette smoking	1.300	0.587-2.877	0.518	0.669	0.074-6.031	0.720
Dyslipidemia	0.718	0.299-1.728	0.460	0.900	0.167-4.846	0.902
Diabetes	1.108	0.141-8.676	0.922	3.074	0.305-30.97	0.341
Visceral obesity	1.180	0.312-4.459	0.808	1.453	0.192-10.98	0.718
**Age group: 30–39 years**	**Univariate analysis**			**Multivariate analysis**		
**Variable**	**Crude HR**	**95% CI**	**p-value**	**Adjusted HR**	**95% CI**	**p-value**
1 year ≤ TTI < 2 years	0.896	0.6081.319	0.577	0.921	0.566-1.499	0.741
TTI ≥ 2 years	0.923	0.6551.301	0.646	1.010	0.648-1.574	0.965
Age	0.992	0.9411.045	0.759	0.986	0.919-1.059	0.704
Male sex	1.370	0.9431.990	0.099	1.261	0.773-2.057	0.354
Systolic blood pressure	1.020	1.0121.028	0.000	1.017	1.007-1.027	0.001
Cigarette smoking	1.201	0.9221.563	0.174	1.339	0.954-1.879	0.091
Dyslipidemia	1.374	1.0371.821	0.027	1.237	0.856-1.789	0.258
Diabetes	2.199	1.4433.350	<0.001	1.915	1.217-3.013	0.005
Visceral obesity	1.642	1.1802.285	0.003	1.154	0.768-1.736	0.490
**Age group: 40–49 years**	**Univariate analysis**			**Multivariate analysis**		

Variable	Crude HR	95% CI	p-value	Adjusted HR	95% CI	p-value
1 year ≤ TTI < 2 years	1.065	0.920-1.234	0.397	1.052	0.890-1.243	0.551
TTI ≥ 2 years	1.143	1.002-1.304	0.047	1.217	1.047-1.415	0.011
Age	1.008	0.992-1.025	0.318	1.014	0.995-1.033	0.146
Male sex	1.505	1.324-1.711	<0.001	1.382	1.186-1.610	0.000
Systolic blood pressure	1.016	1.013-1.020	<0.001	1.017	1.013-1.020	0.000
Cigarette smoking	1.527	1.386-1.683	<0.001	1.346	1.201-1.508	0.000
Dyslipidemia	1.065	0.966-1.174	0.206	0.944	0.843-1.057	0.320
Diabetes	1.454	1.242-1.702	<0.001	1.312	1.114-1.545	0.001
Visceral obesity	1.294	1.168-1.434	<0.001	1.140	1.008-1.288	0.037
**Age group: 50–59 years**	**Univariate analysis**			**Multivariate analysis**		
**Variable**	**Crude HR**	**95% CI**	**p-value**	**Adjusted HR**	**95% CI**	**p-value**
1 year ≤ TTI < 2 years	1.158	1.024-1.309	0.019	1.118	0.9731.285	0.114
TTI ≥ 2 years	1.210	1.080-1.355	0.001	1.258	1.107-1.428	<0.001
Age	1.049	1.035-1.063	<0.001	1.048	1.033-1.064	<0.001
Male sex	1.768	1.598-1.955	<0.001	1.655	1.466-1.868	<0.001
Systolic blood pressure	1.010	1.008-1.013	<0.001	1.011	1.008-1.014	<0.001
Cigarette smoking	1.476	1.364-1.596	<0.001	1.328	1.214-1.453	<0.001
Dyslipidemia	1.091	1.011-1.177	0.025	1.015	0.932-1.106	0.727
Diabetes	1.512	1.357-1.684	<0.001	1.359	1.217-1.519	<0.001
Visceral obesity	1.246	1.153-1.346	<0.001	1.119	1.023-1.225	0.015
**Age group: ≥60 years**	**Univariate analysis**			**Multivariate analysis**		
**Variable**	**Crude HR**	**95% CI**	**p-value**	**Adjusted HR**	**95% CI**	**p-value**
1 year ≤ TTI < 2 years	1.253	1.056-1.485	0.010	1.273	1.0531.538	0.012
TTI ≥ 2 years	1.396	1.181-1.651	<0.001	1.526	1.274-1.828	<0.001
Age	1.061	1.047-1.075	<0.001	1.074	1.058-1.090	<0.001

Male sex	1.407	1.265-1.565	<0.001	1.429	1.257-1.623	<0.001
Systolic blood pressure	1.007	1.004-1.011	<0.001	1.006	1.002-1.010	0.002
Cigarette smoking	1.548	1.397-1.714	<0.001	1.510	1.343-1.698	<0.001
Dyslipidemia	0.988	0.901-1.083	0.796	0.998	0.902-1.105	0.974
Diabetes	1.305	1.149-1.483	<0.001	1.232	1.082-1.403	0.002
Visceral obesity	1.124	1.025-1.233	0.013	1.056	0.951-1.174	0.307

Multivariate Cox regression analysis for predictors of primary outcomes was conducted after adjustment by time to treatment initiation groups, age, male sex, systolic blood pressure, cigarette smoking, dyslipidemia, diabetes, and visceral obesity. HR: hazard ratio; CI: confidence index; TTI: time to treatment initiation.

Supplementary Table 7. Sensitivity analysis: multivariate Cox proportional analysis in a cohort of participants with hypertension detected in examinations after the first physical examination

**Age group: 20–29 years**	**Univariate analysis**			**Multivariate analysis**		
**Variable**	**Crude HR**	**95% CI**	**p-value**	**Adjusted HR**	**95% CI**	**p-value**
1 year ≤ TTI < 2 years	2.938	0.180-48.00	0.450	-	-	-
TTI ≥ 2 years	2.798	0.296-26.43	0.369	-	-	-
Age	0.903	0.717-1.137	0.385	-	-	-
Male sex	-	-	-	-	-	-
Systolic blood pressure	1.031	0.969-1.098	0.332	-	-	-
Cigarette smoking	2.206	0.631-7.712	0.215	-	-	-
Dyslipidemia	3.206	0.535-19.20	0.202	-	-	-
Diabetes	-	-	-	-	-	-
Visceral obesity	-	-	-	-	-	-
**Age group: 30–39 years**	**Univariate analysis**			**Multivariate analysis**		
**Variable**	**Crude HR**	**95% CI**	**p-value**	**Adjusted HR**	**95% CI**	**p-value**
1 year ≤ TTI < 2 years	0.954	0.368-2.473	0.922	1.152	0.3923.379	0.797
TTI ≥ 2 years	1.475	0.661-3.293	0.343	1.386	0.522-3.682	0.512
Age	1.008	0.915-1.111	0.870	1.052	0.912-1.213	0.489
Male sex	0.761	0.452-1.282	0.305	0.844	0.401-1.775	0.654
Systolic blood pressure	0.978	0.952-1.004	0.102	0.984	0.953-1.017	0.339
Cigarette smoking	1.217	0.726-2.039	0.456	1.130	0.561-2.280	0.732
Dyslipidemia	0.888	0.534-1.478	0.649	1.276	0.650-2.503	0.478
Diabetes	1.574	0.482-5.135	0.452	1.742	0.503-6.029	0.381
Visceral obesity	1.090	0.618-1.921	0.766	0.996	0.486-2.038	0.990
**Age group: 40-49 years**	**Univariate analysis**			**Multivariate analysis**		
**Variable**	**Crude HR**	**95% CI**	**p-value**	**Adjusted HR**	**95% CI**	**p-value**

1 year ≤ TTI < 2 years	1.628	1.226-2.163	0.001	1.621	1.190-2.207	0.002
TTI ≥ 2 years	1.313	1.020-1.689	0.034	1.245	0.944-1.642	0.199
Age	1.039	1.005-1.074	0.025	1.051	1.013-1.090	0.008
Male sex	1.218	0.981-1.512	0.074	1.203	0.926-1.563	0.166
Systolic blood pressure	0.999	0.990-1.008	0.853	0.998	0.987-1.008	0.642
Cigarette smoking	1.475	1.224-1.778	0.000	1.353	1.091-1.677	0.006
Dyslipidemia	1.053	0.877-1.265	0.582	1.029	0.836-1.267	0.787
Diabetes	1.126	0.788-1.610	0.514	1.101	0.764-1.586	0.606
Visceral obesity	1.009	0.839-1.214	0.923	0.940	0.754-1.172	0.582

**Age group: 50–59 years**	**Univariate analysis**			**Multivariate analysis**		
**Variable**	**Crude HR**	**95% CI**	**p-value**	**Adjusted HR**	**95% CI**	**p-value**
1 year ≤ TTI < 2 years	1.856	1.470-2.342	<0.001	1.969	1.523-2.547	<0.001
TTI ≥ 2 years	1.711	1.390-2.106	<0.001	1.822	1.447-2.294	<0.001
Age	1.038	1.012-1.064	0.004	1.052	1.023-1.082	<0.001
Male sex	1.763	1.463-2.123	<0.001	1.738	1.389-2.175	<0.001
Systolic blood pressure	1.000	0.993-1.007	0.960	1.000	0.993-1.008	0.965
Cigarette smoking	1.271	1.097-1.473	0.001	1.121	0.950-1.323	0.175
Dyslipidemia	1.134	0.986-1.305	0.079	1.012	0.866-1.181	0.885
Diabetes	1.134	0.903-1.424	0.280	1.053	0.833-1.330	0.667
Visceral obesity	1.258	1.091-1.450	0.002	1.232	1.044-1.455	0.014

**Age group: ≥60 years**	**Univariate analysis**			**Multivariate analysis**		
**Variable**	**Crude HR**	**95% CI**	**p-value**	**Adjusted HR**	**95% CI**	**p-value**
1 year ≤ TTI < 2 years	1.305	0.8901.914	0.173	1.346	0.8832.052	0.167
TTI ≥ 2 years	1.597	1.1472.224	0.006	1.677	1.168-2.407	0.005
Age	1.081	1.0561.107	<0.001	1.095	1.067-1.124	<0.001
Male sex	1.521	1.2421.864	<0.001	1.569	1.239-1.987	<0.001
Systolic blood pressure	1.010	1.0021.019	0.015	1.007	0.998-1.016	0.141
Cigarette smoking	1.092	0.8951.334	0.385	1.047	0.835-1.312	0.691

Dyslipidemia	1.127	0.9501.338	0.170	1.091	0.905-1.316	0.362
Diabetes	1.278	0.9951.642	0.055	1.193	0.924-1.540	0.175
Visceral obesity	1.184	0.9971.407	0.054	1.151	0.945-1.400	0.162

Multivariate Cox regression analysis for predictors of primary outcomes was conducted after adjustment by time to treatment initiation groups, age, male sex, systolic blood pressure, cigarette smoking, dyslipidemia, diabetes, and visceral obesity. HR: hazard ratio; CI: confidence index; TTI: time to treatment initiation.

Supplementary Table 8. Sensitivity analysis: multivariate Cox proportional analysis performed by changing the covariate from visceral obesity (waist circumference) to overweight (body mass index)

**Age group: 20–29 years**	**Univariate analysis**			**Multivariate analysis**		
**Variable**	**Crude HR**	**95% CI**	***p*-value**	**Adjusted HR**	**95% CI**	***p*-value**
1 year ≤ TTI < 2 years	2.482	0.912-6.756	0.075	2.135	0.401-1.332	0.373
TTI ≥ 2 years	1.596	0.616-4.130	0.336	2.872	0.599-13.8	0.187
Age	1.022	0.903-1.157	0.727	1.072	0.883-1.301	0.483
Male sex	1.359	0.605-3.055	0.458	0.684	0.259-1.809	0.444
Systolic blood pressure	1.027	1.009-1.046	0.004	1.013	0.983-1.045	0.393
Cigarette smoking	1.093	0.576-2.074	0.786	0.635	0.208-1.941	0.426
Dyslipidemia	1.455	0.725-2.921	0.291	1.667	0.630-4.406	0.303
Diabetes	0.901	0.121-6.707	0.919	0.881	0.111-6.966	0.904
Overweight	0.920	0.501.-6775	0.786	0.849	0.312-2.310	0.749
**Age group: 30–39 years**	**Univariate analysis**			**Multivariate analysis**		
**Variable**	**Crude HR**	**95% CI**	***p*-value**	**Adjusted HR**	**95% CI**	***p*-value**
1 year ≤ TTI < 2 years	1.001	0.738-1.358	0.997	0.990	0.688-1.424	0.958
TTI ≥ 2 years	1.166	0.896-1.516	0.254	1.286	0.940-1.759	0.115
Age	0.995	0.958-1.034	0.811	0.983	0.939-1.030	0.473
Male sex	1.251	0.976-1.604	0.077	1.035	0.770-1.391	0.818
Systolic blood pressure	1.010	1.004-1.016	0.001	1.005	0.997-1.012	0.199
Cigarette smoking	1.200	0.986-1.460	0.069	1.228	0.969-1.555	0.089
Dyslipidemia	1.341	1.097-1.638	0.004	1.377	1.073-1.767	0.012
Diabetes	1.877	1.335-2.641	<0.001	1.834	1.289-2.612	0.001
Overweight	1.107	0.907-1.352	0.316	0.872	0.680-1.119	0.283

Age group: 40–49 years	Univariate analysis			Multivariate analysis		
**Variable**	**Crude HR**	**95% CI**	***p*-value**	**Adjusted HR**	**95% CI**	***p*-value**
1 year ≤ TTI < 2 years	1.228	1.099-1.372	<0.001	1.201	1.061-1.359	0.004
TTI ≥ 2 years	1.243	1.129-1.368	<0.001	1.281	1.150-1.427	<0.001
Age	1.016	1.004-1.029	0.011	1.021	1.007-1.035	0.003
Male sex	1.343	1.227-1.471	<0.001	1.262	1.136-1.402	<0.001
Systolic blood pressure	1.011	1.009-1.014	<0.001	1.011	1.008-1.013	<0.001
Cigarette smoking	1.450	1.349-1.558	<0.001	1.356	1.248-1.474	<0.001
Dyslipidemia	1.103	1.027-1.185	0.007	1.015	0.935-1.102	0.721
Diabetes	1.484	1.319-1.670	<0.001	1.369	1.212-1.546	<0.001
Overweight	1.174	1.093-1.262	<0.001	1.085	0.998-1.180	0.054

Age group: 50–59 years	Univariate analysis			Multivariate analysis		
**Variable**	**Crude HR**	**95% CI**	***p*-value**	**Adjusted HR**	**95% CI**	***p*-value**
1 year ≤ TTI < 2 years	1.295	1.180-1.420	<0.001	1.278	1.154-1.415	<0.001
TTI ≥ 2 years	1.299	1.197-1.410	<0.001	1.320	1.206-1.445	<0.001
Age	1.046	1.035-1.056	<0.001	1.048	1.037-1.060	<0.001
Male sex	1.707	1.587-1.837	<0.001	1.616	1.486-1.756	<0.001
Systolic blood pressure	1.008	1.006-1.010	<0.001	1.008	1.006-1.010	<0.001
Cigarette smoking	1.419	1.340-1.503	<0.001	1.288	1.207-1.374	<0.001
Dyslipidemia	1.108	1.049-1.171	<0.001	1.030	0.969-1.095	0.343
Diabetes	1.463	1.349-1.586	<0.001	1.332	1.226-1.447	<0.001
Overweight	1.092	1.034-1.154	0.002	1.066	1.002-1.134	0.043

Age group: ≥60 years	Univariate analysis			Multivariate analysis		
**Variable**	**Crude HR**	**95% CI**	***p*-value**	**Adjusted HR**	**95% CI**	***p*-value**
1 year ≤ TTI < 2 years	1.236	1.081-1.414	0.002	1.215	1.047-1.410	0.010

TTI ≥ 2 years	1.426	1.263-1.609	<0.001	1.502	1.318-1.711	<0.001
Age	1.069	1.058-1.079	<0.001	1.081	1.070-1.093	<0.001
Male sex	1.377	1.274-1.488	<0.001	1.398	1.280-1.528	<0.001
Systolic blood pressure	1.007	1.005-1.009	<0.001	1.006	1.003-1.008	<0.001
Cigarette smoking	1.453	1.348-1.567	<0.001	1.438	1.321-1.566	<0.001
Dyslipidemia	1.056	0.988-1.129	0.111	1.046	0.972-1.125	0.231
Diabetes	1.229	1.116-1.354	<0.001	1.139	1.032-1.256	0.009
Overweight	1.135	1.060-1.215	<0.001	1.184	1.098-1.276	<0.001

Multivariate Cox regression analysis for predictors of primary outcomes was conducted with adjustment by time to treatment initiation groups, age, male sex, systolic blood pressure, cigarette smoking, dyslipidemia, diabetes, and overweight. HR: hazard ratio; CI: confidence index; TTI: time to treatment initiation.

Supplementary Table 9. Sensitivity analysis: multivariate Cox proportional analysis after multiple imputation for missing data.

**Age group: 20–29 years**	**Univariate analysis**			**Multivariate analysis**		
**Variable**	**Crude HR**	**95% CI**	***p*-value**	**Adjusted HR**	**95% CI**	***p*-value**
1 year ≤ TTI < 2 years	1.955	1.605-2.381	<0.001	1.938	1.591-2.361	<0.001
TTI ≥ 2 years	1.868	1.602-2.178	<0.001	2.110	1.804-2.467	<0.001
Age	1.021	0.993-1.050	0.140	1.032	1.003-1.061	0.029
Male sex	1.363	1.137-1.633	0.001	1.264	1.044-1.531	0.016
Systolic blood pressure	1.023	1.019-1.027	<0.001	1.026	1.022-1.031	<0.001
Cigarette smoking	1.139	0.987-1.313	0.075	1.015	0.878-1.174	0.837
Dyslipidemia	1.229	1.075-1.405	0.003	1.088	0.938-1.263	0.264
Diabetes	1.102	0.830-1.463	0.503	0.990	0.740-1.324	0.944
Visceral obesity	1.065	0.931-1.219	0.360	0.790	0.677-0.923	0.003
**Age group: 30–39 years**	**Univariate analysis**			**Multivariate analysis**		
**Variable**	**Crude HR**	**95% CI**	***p*-value**	**Adjusted HR**	**95% CI**	***p*-value**
1 year ≤ TTI < 2 years	1.033	0.967-1.103	0.334	1.081	1.012-1.155	0.021
TTI ≥ 2 years	1.185	1.132-1.242	<0.001	1.305	1.244-1.369	<0.001
Age	0.998	0.989-1.006	0.611	0.998	0.990-1.007	0.707
Male sex	1.267	1.198-1.339	<0.001	1.089	1.026-1.156	0.005
Systolic blood pressure	1.009	1.008-1.011	<0.001	1.008	1.006-1.009	<0.001
Cigarette smoking	1.203	1.151-1.257	<0.001	1.116	1.067-1.168	<0.001
Dyslipidemia	1.334	1.276-1.394	<0.001	1.189	1.134-1.246	<0.001
Diabetes	1.952	1.834-2.078	<0.001	1.829	1.715-1.952	<0.001
Visceral obesity	1.270	1.214-1.329	<0.001	1.080	1.027-1.135	0.003
**Age group: 40–49 years**	**Univariate analysis**			**Multivariate analysis**		

Variable	Crude HR	95% CI	*p*-value	Adjusted HR	95% CI	*p*-value
1 year ≤ TTI < 2 years	1.232	1.203-1.262	<0.001	1.241	1.211-1.271	<0.001
TTI ≥ 2 years	1.244	1.222-1.267	<0.001	1.329	1.305-1.354	<0.001
Age	1.041	1.012-1.017	<0.001	1.022	1.020-1.025	<0.001
Male sex	1.368	1.340-1.396	<0.001	1.244	1.217-1.272	<0.001
Systolic blood pressure	1.011	1.011-1.012	<0.001	1.012	1.011-1.012	<0.001
Cigarette smoking	1.456	1.433-1.480	<0.001	1.357	1.335-1.380	<0.001
Dyslipidemia	1.105	1.087-1.123	<0.001	0.982	0.966-0.999	0.033
Diabetes	1.500	1.465-1.536	<0.001	1.414	1.380-1.448	<0.001
Visceral obesity	1.205	1.185-1.224	<0.001	1.097	1.078-1.117	<0.001
**Age group: 50–59 years**	**Univariate analysis**			**Multivariate analysis**		
**Variable**	**Crude HR**	**95% CI**	***p*-value**	**Adjusted HR**	**95% CI**	***p*-value**
1 year ≤ TTI < 2 years	1.360	1.332-1.387	<0.001	1.367	1.340-1.396	<0.001
TTI ≥ 2 years	1.488	1.464-1.513	<0.001	1.545	1.520-1.571	<0.001
Age	1.040	1.038-1.042	<0.001	1.048	1.046-1.050	<0.001
Male sex	1.745	1.717-1.774	<0.001	1.575	1.548-1.603	<0.001
Systolic blood pressure	1.008	1.008-1.009	<0.001	1.008	1.008-1.009	<0.001
Cigarette smoking	1.434	1.416-1.453	<0.001	1.270	1.253-1.286	<0.001
Dyslipidemia	1.112	1.098-1.126	<0.001	1.036	1.023-1.049	<0.001
Diabetes	1.428	1.404-1.452	<0.001	1.317	1.295-1.340	<0.001
Visceral obesity	1.241	1.149-1.184	<0.001	1.107	1.093-1.122	<0.001
**Age group: ≥60 years**	**Univariate analysis**			**Multivariate analysis**		
**Variable**	**Crude HR**	**95% CI**	***p*-value**	**Adjusted HR**	**95% CI**	***p*-value**
1 year ≤ TTI < 2 years	1.385	1.345-1.427	<0.001	1.439	1.397-1.483	<0.001
TTI ≥ 2 years	1.876	1.830-1.923	<0.001	2.037	1.987-2.089	<0.001
Age	1.062	1.038-1.042	<0.001	1.079	1.076-1.081	<0.001

Male sex	1.389	1.365-1.413	<0.001	1.331	1.306-1.356	<0.001
Systolic blood pressure	1.007	1.006-1.007	<0.001	1.006	1.005-1.006	<0.001
Cigarette smoking	1.466	1.442-1.491	<0.001	1.427	1.402-1.452	<0.001
Dyslipidemia	1.062	1.046-1.078	<0.001	1.026	1.011-1.042	0.001
Diabetes	1.236	1.212-1.261	<0.001	1.174	1.150-1.198	<0.001
Visceral obesity	1.166	1,149-1.184	<0.001	1.117	1.099-1.135	<0.001

Multivariate Cox regression analysis for predictors of primary outcomes was conducted with adjustment by time to treatment initiation groups, age, male sex, systolic blood pressure, cigarette smoking, dyslipidemia, diabetes, and visceral obesity. HR: hazard ratio; CI: confidence index; TTI: time to treatment initiation.

Supplementary Table 10. Sensitivity analysis: multivariate Cox proportional analysis excluding events that occurred within the first year after treatment initiation.

**Age group: 20–29 years**	**Univariate analysis**			**Multivariate analysis**		
**Variable**	**Crude HR**	**95% CI**	***p*-value**	**Adjusted HR**	**95% CI**	***p*-value**
1 year ≤ TTI < 2 years	3.319	0.975-11.30	0.055	-	-	-
TTI ≥ 2 years	2.132	0.878-5.177	0.094	0.608	0.077-4.807	0.637
Age	1.042	0.857-1.267	0.679	0.730	0.468-1.139	0.165
Male sex	1.052	0.358-3.091	0.927			-
Systolic blood pressure	1.013	0.989-1.037	0.300	0.976	0.912-1.046	0.495
Cigarette smoking	0.999	0.432-2.309	0.998	2.225	0.213-23.21	0.504
Dyslipidemia	1.630	0.642-4.143	0.304	-	-	-
Diabetes	-	-	-	-	-	-
Visceral obesity	4.612	0.538-39.56	0.163	0.621	0.038-10.03	0.737

**Age group: 30–39 years**	**Univariate analysis**			**Multivariate analysis**		
**Variable**	**Crude HR**	**95% CI**	***p*-value**	**Adjusted HR**	**95% CI**	***p*-value**
1 year ≤ TTI < 2 years	0.780	0.506-1.202	0.26	0.818	0.472-1.419	0.476
TTI ≥ 2 years	1.255	0.959-1.641	0.098	1.491	1.031-2.157	0.034
Age	0.981	0.933-1.032	0.465	0.959	0.896-1.025	0.219
Male sex	1.227	0.889-1.694	0.213	1.657	1.055-2.604	0.029
Systolic blood pressure	1.000	0.993-1.008	0.902	0.998	0.988-1.008	0.729
Cigarette smoking	0.925	0.715-1.197	0.552	0.846	0.604-1.185	0.331
Dyslipidemia	1.385	1.067-1.798	0.014	1.359	0.949-1.946	0.095
Diabetes	1.092	0.714-1.670	0.686	1.465	0.929-2.311	0.100
Visceral obesity	1.328	0.995-1.773	0.054	1.000	0.687-1.455	0.999

**Age group: 40–49 years**	**Univariate analysis**			**Multivariate analysis**		

Variable	Crude HR	95% CI	*p*-value	Adjusted HR	95% CI	*p*-value
1 year ≤ TTI < 2 years	0.978	0.850-1.126	0.760	0.956	0.817-1.117	0.570
TTI ≥ 2 years	1.255	1.125-1.401	<0.001	1.270	1.120-1.440	<0.001
Age	1.005	0.990-1.021	0.495	1.014	0.997-1.032	0.107
Male sex	1.136	1.013-1.275	0.029	1.079	0.941-1.238	0.275
Systolic blood pressure	1.006	1.004-1.009	<0.001	1.006	1.003-1.010	<0.001
Cigarette smoking	1.192	1.088-1.305	<0.001	1.161	1.045-1.290	0.006
Dyslipidemia	1.023	0.935-1.119	0.619	1.002	0.904-1.110	0.977
Diabetes	1.279	1.115-1.468	<0.001	1.264	1.096-1.458	0.001
Visceral obesity	1.058	0.964-1.162	0.233	0.992	0.888-1.107	0.879
**Age group: 50–59 years**	**Univariate analysis**			**Multivariate analysis**		
**Variable**	**Crude HR**	**95% CI**	***p*-value**	**Adjusted HR**	**95% CI**	***p*-value**
1 year ≤ TTI < 2 years	1.122	0.996-1.264	0.057	1.107	0.968-1.266	0.138
TTI ≥ 2 years	1.270	1.143-1.412	<0.001	1.392	1.236-1.568	<0.001
Age	1.055	1.042-1.067	<0.001	1.063	1.049-1.077	<0.001
Male sex	1.276	1.164-1.398	<0.001	1.221	1.095-1.361	<0.001
Systolic blood pressure	1.004	1.001-1.006	0.002	1.002	0.999-1.004	0.236
Cigarette smoking	1.148	1.070-1.232	<0.001	1.094	1.010-1.185	0.028
Dyslipidemia	1.077	1.007-1.152	0.031	1.040	0.964-1.121	0.311
Diabetes	1.205	1.096-1.325	<0.001	1.180	1.070-1.302	0.001
Visceral obesity	1.106	1.031-1.185	0.005	1.069	0.986-1.159	0.104
**Age group: ≥60 years**	**Univariate analysis**			**Multivariate analysis**		
**Variable**	**Crude HR**	**95% CI**	***p*-value**	**Adjusted HR**	**95% CI**	***p*-value**
1 year ≤ TTI < 2 years	1.291	1.086-1.535	0.004	1.289	1.066-1.559	0.009
TTI ≥ 2 years	1.527	1.285-1.815	<0.001	1.635	1.359-1.967	<0.001
Age	1.051	1.037-1.065	<0.001	1.061	1.046-1.077	<0.001

Male sex	1.197	1.087-1.318	<0.001	1.200	1.070-1.346	0.002
Systolic blood pressure	1.003	1.001-1.006	0.013	1.004	1.001-1.007	0.019
Cigarette smoking	1.320	1.201-1.451	<0.001	1.336	1.201-1.485	<0.001
Dyslipidemia	1.039	0.957-1.129	0.359	1.042	0.952-1.141	0.374
Diabetes	1.090	0.969-1.225	0.152	1.034	0.918-1.166	0.582
Visceral obesity	1.097	1.009-1.193	0.029	1.096	0.996-1.205	0.059

Multivariate Cox regression analysis for predictors of primary outcomes was conducted with adjustment by time to treatment initiation groups, age, male sex, systolic blood pressure, cigarette smoking, dyslipidemia, diabetes, and visceral obesity. HR: hazard ratio; CI: confidence index; TTI: time to treatment initiation.

Supplementary Table 11. Sensitivity analysis: Hazard ratios for the primary endpoints considering competing mortality risks using Fine-Gray modeling

**Age group: 20–29 years**	**Competing risks model**		
**Variable**	**Adjusted HR**	**95% CI**	***p*-value**
1 year ≤ TTI < 2 years	0.928	0.550-1.564	0.780
TTI ≥ 2 years	1.002	0.660-1.521	0.990
Age	0.977	0.905-1.054	0.550
Male sex	0.706	0.440-1.135	0.150
Systolic blood pressure	1.003	0.987-1.018	0.740
Cigarette smoking	0.800	0.496-1.292	0.360
Dyslipidemia	0.955	0.630-1.447	0.830
Diabetes	0.818	0.327-2.045	0.670
Visceral obesity	1.268	0.801-2.005	0.310
**Age group: 30–39 years**	**Competing risks model**		
**Variable**	**Adjusted HR**	**95% CI**	***p*-value**
1 year ≤ TTI < 2 years	1.370	1.195-1.571	<0.001
TTI ≥ 2 years	1.355	1.219-1.507	<0.001
Age	0.995	0.976-1.015	0.640
Male sex	1.155	1.019-1.308	0.024
Systolic blood pressure	1.008	1.005-1.012	<0.001
Cigarette smoking	1.062	0.959-1.177	0.250
Dyslipidemia	1.114	1.006-1.235	0.038
Diabetes	0.972	0.805-1.173	0.770
Visceral obesity	0.954	0.856-1.062	0.390
**Age group: 40–49 years**	**Competing risks model**		
**Variable**	**Adjusted HR**	**95% CI**	**p-value**
1 year ≤ TTI < 2 years	1.415	1.344-1.490	<0.001
TTI ≥ 2 years	1.526	1.470-1.584	<0.001
Age	1.009	1.003-1.015	0.003

Male sex	1.051	1.005-1.098	0.028
Systolic blood pressure	1.006	1.005-1.007	<0.001
Cigarette smoking	1.201	1.157-1.246	<0.001
Dyslipidemia	1.067	1.030-1.105	<0.001
Diabetes	1.137	1.072-1.205	<0.001
Visceral obesity	1.033	0.995-1.073	0.092

**Age group: 50–59 years**	**Competing risks model**		
**Variable**	**Adjusted HR**	**95% CI**	**p-value**
1 year ≤ TTI < 2 years	1.569	1.499-1.643	<0.001
TTI ≥ 2 years	1.801	1.738-1.867	<0.001
Age	1.026	1.021-1.031	<0.001
Male sex	1.135	1.095-1.178	<0.001
Systolic blood pressure	1.004	1.003-1.005	<0.001
Cigarette smoking	1.182	1.145-1.220	<0.001
Dyslipidemia	1.045	1.015-1.075	0.003
Diabetes	1.153	1.106-1.203	<0.001
Visceral obesity	1.059	1.027-1.091	<0.001

**Age group: ≥60 years**	**Competing risks model**		
**Variable**	**Adjusted HR**	**95% CI**	**p-value**
1 year ≤ TTI < 2 years	1.724	1.615-1.841	<0.001
TTI ≥ 2 years	2.107	1.990-2.230	<0.001
Age	1.057	1.052-1.063	<0.001
Male sex	1.171	1.123-1.221	<0.001
Systolic blood pressure	1.002	1.001-1.004	<0.001
Cigarette smoking	1.191	1.141-1.244	<0.001
Dyslipidemia	1.029	0.993-1.066	0.110
Diabetes	1.022	0.972-1.075	0.400
Visceral obesity	1.027	0.990-1.066	0.150

Multivariate Cox regression analysis for predictors of primary outcomes was conducted with adjustment by time to

treatment initiation groups, age, male sex, systolic blood pressure, cigarette smoking, dyslipidemia, diabetes, and visceral obesity. HR: hazard ratio; CI: confidence index; TTI: time to treatment initiation.
